# IgE antibodies increase honeybee venom responsiveness and detoxification efficiency of mast cells

**DOI:** 10.1111/all.14852

**Published:** 2021-05-06

**Authors:** Philipp Starkl, Nicolas Gaudenzio, Thomas Marichal, Laurent L. Reber, Riccardo Sibilano, Martin L. Watzenboeck, Frédéric Fontaine, André C. Mueller, Mindy Tsai, Sylvia Knapp, Stephen J. Galli

**Affiliations:** 1 Laboratory of Infection Biology, Department of Medicine I, Medical University of Vienna, Vienna, Austria; 2 CeMM - Research Center for Molecular Medicine of the Austrian Academy of Sciences, Vienna, Austria; 3 Department of Pathology, Stanford University School of Medicine, Stanford, CA, USA; 4 Toulouse Institute for Infectious and Inflammatory Diseases, INSERM UMR1291, CNRS, UMR5051, University of Toulouse III, Toulouse, France; 5 GIGA-Research and Faculty of Veterinary Medicine, University of Liege, Liege, Belgium; 6 Sean N. Parker Center for Allergy and Asthma Research, Stanford University, Stanford, CA, USA; 7 Department of Biomedical Imaging and Image-guided Therapy, Medical University of Vienna, Vienna, Austria; 8 Department of Microbiology and Immunology, Stanford University School of Medicine, Stanford, CA, USA

**Keywords:** honeybee venom, host defense, IgE, mast cells, toxin hypothesis

## Abstract

**Background:**

In contrast to their clearly defined roles in allergic diseases, the physiologic functions of Immunoglobulin E antibodies (IgEs) and mast cells (MCs) remain enigmatic. Recent research supports the *toxin hypothesis*, showing that MCs and IgE-related type 2 immune responses can enhance host defense against certain noxious substances, including honeybee venom (BV). However, the mechanisms by which MCs can interfere with BV toxicity are unknown. In this study, we assessed the role of IgE and certain MC products in MC-mediated BV detoxification.

**Methods:**

We applied *in vitro* and *in vivo* fluorescence microscopyimaging, and flow cytometry, fibroblast-based toxicity assays and mass spectrometry to investigate IgE-mediated detoxification of BV cytotoxicity by mouse and human MCs *in vitro*. Pharmacologic strategies to interfere with MC-derived heparin and proteases helped to define the importance of specific detoxification mechanisms.

**Results:**

Venom-specific IgE increased the degranulation and cytokine responses of MCs to BV*in vitro*. Passive serum sensitization enhanced MC degranulation *in vivo*. IgE-activated mouse or human MCs exhibited enhanced potential for detoxifying BV by both proteolytic degradation and heparin-related interference with toxicity. Mediators released by IgE-activated human MCs efficiently degraded multiple BV toxins.

**Conclusions:**

Our results both reveal that IgE sensitization enhances the MC’s ability to detoxify BV and also assign efficient toxin-neutralizing activity to MC-derived heparin and proteases. Our study thus highlights the potential importance of IgE, MCs, and particular MC products in defense against BV.

## GRAPHICAL ABSTRACT

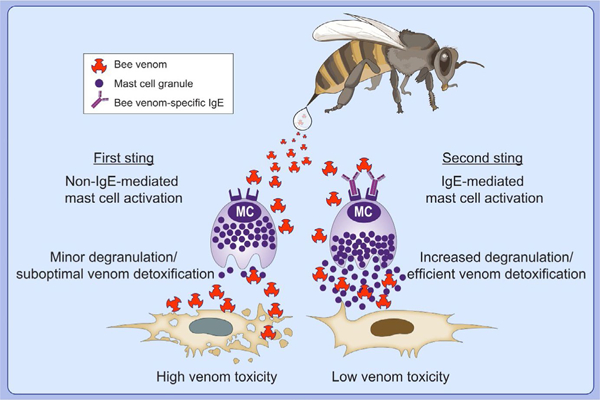

Immunoglobulin E antibodies increase mast cell responsiveness to honeybee venom *in vitro* and *in vivo*. Compounds released by IgE-activated mouse and human mast cells can efficiently detoxify honeybee venom. Mast cell-derived heparin and proteases contribute to prevention of honeybee venom toxicity

## 1 | BACKGROUND

Mast cells (MCs) and immunoglobulin E antibodies (IgEs) are generally best known for their adverse functions in allergic diseases.^1^ Allergic patients elaborate IgEs recognizing epitopes on environmental antigens, which typically represent no obvious danger. However, the exposure of sensitized hosts to antigens recognized by IgEs bound to MCs via the high-affinity IgE receptor, FcεRI, result in rapid MC activation with the release of many bioactive compounds, including MC-associated proteases and proteoglycans(eg, heparin), as well as diverse additional stored and newly synthesized mediators.^1,2^ In concert, such MC-derived mediators cause immediate allergic symptoms and inflammation,^1,2^ in extreme cases leading to potentially fatal anaphylaxis.^3^

The evolutionary conservation of IgE and MCs despite their life-threatening potential could reflect important protective functions requiring fast and strong immunological and behavioral responses.^4^ Traditionally, IgE and MCs have been assigned a protective role in host defense against parasites.^4–6^ However, the importance of such protective functions seems to depend on the respective parasite and model system.^5^ In contrast to parasites, toxins and venoms represent acute and highly dangerous threats that require immediate counter-measures in order to avoid extensive damage,^7^ and it has been suggested that allergies developed initially as protective responses to noxious substances.^8–10^

Notably, several venoms either contain or are themselves allergens.^8,9^ One example of a highly allergenic venom is honeybee (*Apis mellifera*) venom (BV).^11,12^ BV is a complex mixture of active substances, including neurotoxins (such as apamin), enzymes (such as phospholipase A2 [PLA_2_]), amines, and the cytolytic peptidemelittin.^13^ PLA_2_, hyaluronidase, acid phosphatase, and melittin are considered the main BV allergens (designated Api m1–4, respectively).^11^ Many BV constituents induce acute pain upon injection into the opponent’s tissue.^13^ In addition, BV contains compounds which can induce MC degranulation.

Recent evidence shows that mouse MCs can play an important role in innate host defense against an initial challenge with various venoms, including those of different reptiles,^14–16^ scorpions^16^ and the honeybee.^14^ These studies revealed that detoxification of snake, Gila monster, and scorpion venoms was dependent, in large part, on the proteolytic activity of the MC-specific proteases carboxypeptidase A3^14,15^ (CPA3, in case of certain snake venoms) and the chymasemMCP4^16^ (for Gila monster and scorpion venoms). Moreover, for the venoms of the Russell’s viper^17^ and the honeybee,^18,19^ we^17,18^ and others^19^ showed that subcutaneous sublethal injections of venom induce a type 2 immune response associated with the production of specific IgEs, and that such IgEs and IgE effector cells can play important roles in acquired immunity against subsequent exposure to potentially lethal amounts of the venom^17,18^ or one of its constituents.^19^ Yet the specific identity of MC-associated defenses against BV remained unclear.

Thus, while it is established that MCs and IgEs can be important in host defense against BV,^5^ the mechanisms by which IgE enhances MC-mediated detoxification of BV have not been clarified. In addition, the MC signal transduction response to BV in the absence or presence of IgE is uncharacterized. Here, we performed studies on primary mouse and human MCs to decipher potential mechanisms of BV detoxification.

## 2 | METHODS

Additional methodological details regarding statistics, cellular models, analyses of gene expression, signal transduction, degranulation, viability, degradation of BV, proteomics, PLA_2_ activity, imaging, and venom cytotoxicity, as well as more information regarding the mice, reagents, and antibodies used in this study, can be found in the online Appendix S1.

### 2.1 | Mice

Animal care and experiments were performed following current guidelines of the National Institutes of Health and with approval of the Stanford University Institutional Animal Care and Use Committee (IACUC protocol #12683). C57BL/6 wildtype (wt) mice were obtained from Jackson Laboratories and housed in the Stanford University animal facility for at least 7 days before starting experiments.

### 2.2 | Serum generation

For generation of mouse sera, C57BL/6 wtmice were shaved on the back skin and received two subcutaneous injections of 50 µl (containing 200 µg) BV (Lot 12071006HB; ALK Abello Source Material) or PBS in the shaved area.^18^ Three weeks later, mice were sacrificed and sera were collected.

### 2.3 | Statistics

Statistical analyses were conducted using GraphPad Prism (GraphPad Software), and tests were applied as indicated in the figure legends. Differences with *p* values equal or below 0.05 were considered significant. All experiments were performed at least twice.

## 3 | RESULTS

### 3.1 | IgE sensitization increases BV-mediated MC degranulation and signaling

**FIGURE 1:**
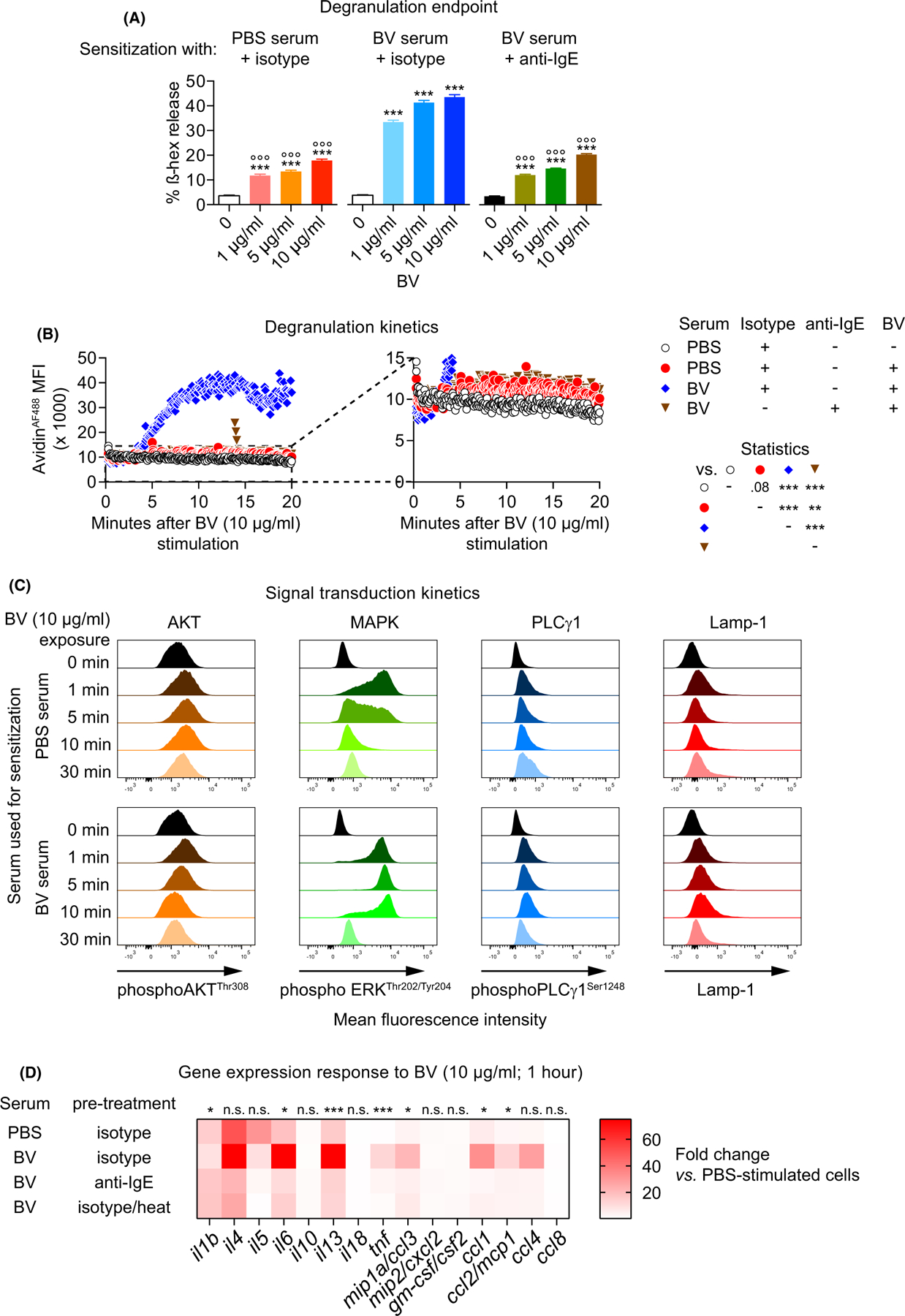
Immunoglobulin E sensitization modulates FSCMC degranulation, signaling, and gene expression responses to honeybee venom. FSCMCs were sensitized overnight with serum derived either from mock (PBS)-immunized or BV-immunized mice (PBS serum or BV serum, respectively) that was either untreated or pre-treated (before cell incubation) with (A, B, D) an anti-IgE (or isotype control) antibody or (D) by heating as indicated. (A) FSCMCs were unstimulated or exposed to BV at 1, 5, or 10 µg/ml for 1 h and analyzed for ß hexosaminidase (ß-hex) release (data are from one of three independent experiments, each of which gave similar results). (B) FSCMCs were stimulated with 10 µg/ml BV and fluorescent signal in live cells due to heparin/avidin^AF488^ interaction was recorded over 20 min by flow cytometry. The avidin^AF488^ mean fluorescence intensity (MFI) kinetics are shown. The right panel represents a y-axis magnification of the data as indicated by the dashed lines in the left graph in order to allow identification of minor fluorescence differences (data are from one of three independent experiments, each of which gave similar results). (C) Phosphorylation of AKT, MAPK/ERK and PLCγ1 and membrane-localized Lamp-1 was analyzed in FSCMCs without stimulation (0 min) and after exposure to 10 µg BV for 1, 5, 10, or 30 min by (phospho-) flow cytometry (representative of three independent experiments). (D) Heatmap representing gene expression data (mean of triplicates) from realtime PCR analysis of cytokines and chemokines in FSCMCs after 1 h exposure to 10 µg/ml BV (fold change compared to non-stimulated cells; data are from one of two independent experiments, each of which gave similar results).(A and B) Two-way ANOVA with Tukey’s test for multiple comparisons; (A) * indicates comparisons to the respective unstimulated ctrl in the group; ° indicates comparisons with the same concentration of the BV serum group; *p* values are adjusted for multiple testing; graphs represent mean +SD; (D) * (or n.s.—not significant) indicates comparisons of the fold changes of PBS serum/isotype-treated vs. BV serum/isotype-treated groups by *t* test (neither BV serum/anti-IgE-treated nor BV serum/heat-treated groups were significantly different compared to the PBS serum/isotype-treated samples for any of the investigated genes); ns: not significant;*/°*p* ≤ .05; **/°°*p* ≤ .01; ***/°°°*p* ≤ .001; numbers in (A) show the actual *p* value

We first tested the ability of BV to cause degranulation and cytotoxicity in mouse fetal skin-derived cultured MCs (FSCMCs). Assessing degranulation by detecting release of the granule enzyme ß-hexosaminidase, and monitoring viability by flow cytometry, we found significant BV-related degranulation of FSCMCs at 1 or 10 µg/ml BV without detectable effects on cell viability (Figure S1A,B). To dissect the effect of sensitization with specific IgE on FSCMC responsiveness to BV, we generated immune serum collected from mice 3 weeks after two subcutaneous injections of either 200 µg BV (BV serum) or the respective amount of PBS (PBS serum). This treatment, that approximately mimicked the dose of venom injected by two bee stings,^18,20^ induced elevated levels of serum IgE, IgG1, and IgG2b (Figure S1C). Sensitization with BV serum significantly increased FSCMC degranulation at all tested sub-cytotoxic doses (1, 5, or 10 µg/ml), and pre-treatment of BV serum with anti-IgE almost completely attenuated this effect (Figure 1A).

We then used flow cytometry to monitor FSCMC degranulation in real-time, assessing degranulation by fluorescence emitted upon binding of Alexa Fluor 488-tagged avidin [avidin^AF488^] to granular heparin exposed on the cell surface upon granule exocytosis.^21^ We found that degranulation of BVserum-sensitizedFSCMCs, which began less than 5 min after addition of BV, reached a peak plateau after 10–15 min (Figure 1B). Anti-IgE pre-treatment again abrogated degranulation to a level indistinguishable to that of cells mock-sensitized with PBS serum (Figure 1B).

These results indicate that subcutaneous injections of BV induce specific IgE antibodies that strongly amplified the innate degranulation response of FSCMCs to BV. To investigate this further, we analyzed the effect of serum sensitization on BV-related FSCMC signaling. Kinetic analysis of major signaling pathways transducing MC activation signals^22^ revealed that BV exposure increased phosphorylation of AKT, ERK, and PLCγ in FSCMCs, indicating IgE-independent transduction of activation in these PBS serum-sensitized FSCMCs (Figure 1C). Interestingly, sensitization with BV serum markedly increased BV-mediated ERK phosphorylation (Figure 1C).

The mediator release response of MCs can be tuned depending on the stimulation signals.^23,24^ For instance, IgE/antigen-mediated activation via FcεRI leads to degranulation and extensive cytokine production, whereas stimulation viaG protein-coupled receptors, such as Mrgprb2, induces predominantly degranulation with minor*de novo* cytokine production.^24,25^ We therefore wanted to test whether IgE sensitization affected mediator synthesis of BV-stimulated MCs. We observed that BV exposure induced increased transcription of *il1b*, *il4*, *il5*, *il6*, *il13*, *mip1a*, *ccl1*, *ccl2*, and*ccl4* (Figure 1D). However, many elements of this innate response were profoundly amplified by sensitization with untreated BV serum, reflected by increased levels of *il1b*, *il6*, *il13*, *tnf*, *mip1a*, *ccl1*, and *ccl2*. Interference with IgE function, either by serum pre-treatment with anti-IgE or heating the serum to 56C°,^18^ largely abolished the increased cytokine and chemokine transcription, indicating the important contribution of IgE (Figure 1D).

**FIGURE 2:**
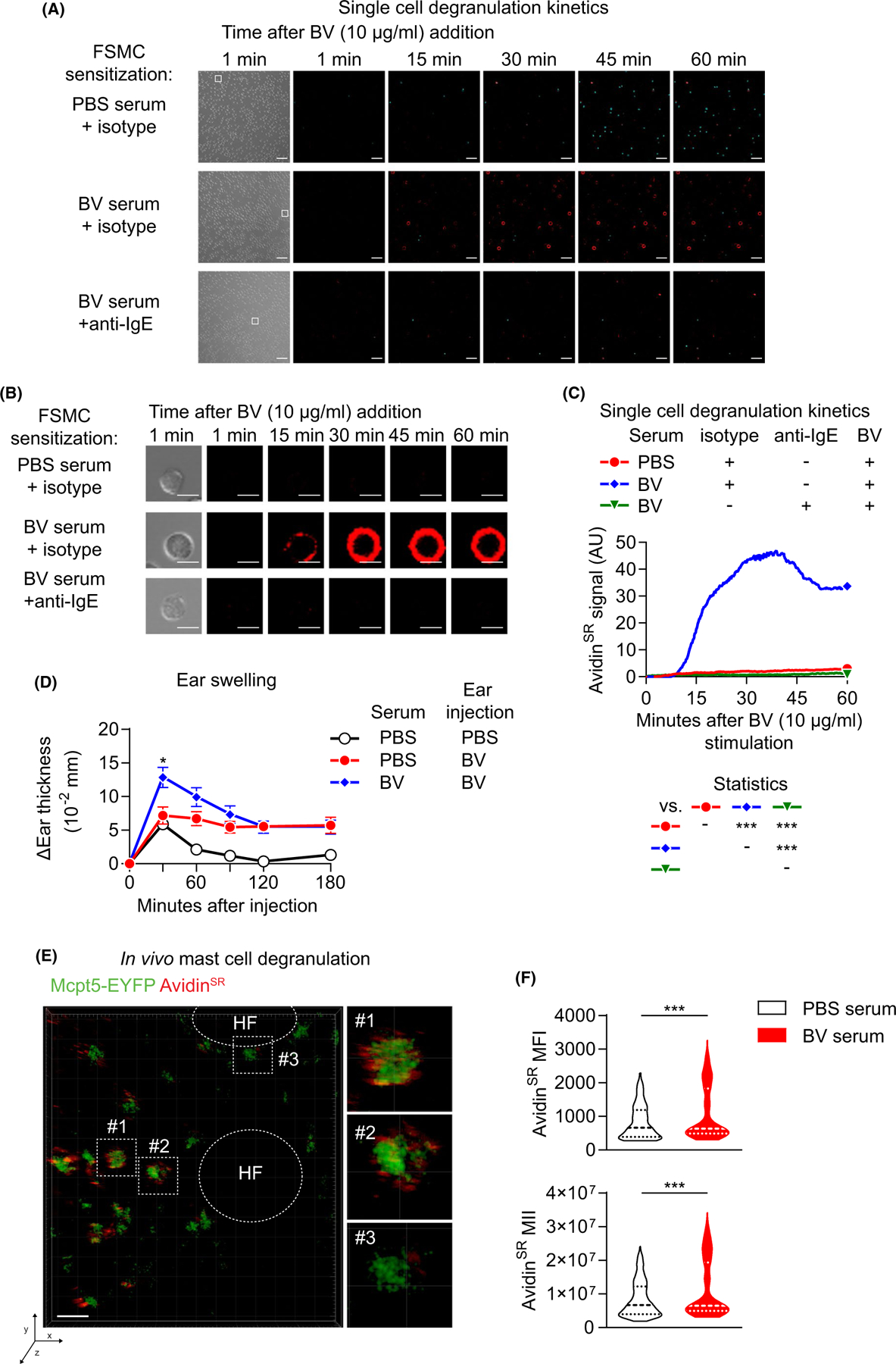
Serum sensitization increases FSCMC activation at the single-cell level. (A–C) FSCMCs were incubated overnight with isotype control-pre-treated PBS serumor isotype control- or anti-IgE-pre-treated BV serum. Next day, cells were seeded in chamber slides and exposed to 10 µg/ml BV. The avidin^SR^-mediated signal of degranulating MCs was recorded over 60 min by realtime confocal fluorescence microscopy. (A) Snap shots of brightfield (outermost left) 1 min and fluorescence signals (merge of To-Pro-3 [viability, in turquoise] and avidin^SR^ [in red]) at the indicated time points after BV addition are shown. The white squares in the 1 min brightfield picture indicate the individual cells presented in (B).(B) Brightfield and immunofluorescence pictures of individual representative cells from (A) (as indicated by the white squares) at 1, 15,30, 45, and 60 min after BV exposure are shown. The scale bars in (A) and (B) represent 10 µm. (C) quantification of the mean avidin^SR^ signal kinetics of cells in field of views shown in (A). (D–F) C57BL/6 mice were intradermally injected with either PBS serum or BV serum into both ears. Next day, one ear of each mouse was injected with either 200 ng BV or PBS. (D) Ear thickness measured directly before and at indicated timepoints after injection. (E and F) *Mcpt5-Cre; R26Y*^*EYFP*^ mice were pre-treated with immune sera as described for (D). The next day, the serum-treated ear of each mouse was intradermally injected with 200 ng BV containing 8 µg avidin^SR^. Thirty minutes after injection, the fluorescent avidin^SR^signal within a circular area of 60 µm surrounding single EYFP^+^ cells wasdetermined by intravital 2-photon microscopy. (E) The large picture shows a representative intradermal area of the ear, with EYFP (green, dermal MCs) and avidin^SR^ signal (red, exteriorized MC cytoplasmic granules) indicated (scale barsrepresent30 µm). The smaller pictures on the right represent magnifications of individual MCs as indicated in the large picture by squares with dashed lines. HF indicates hair follicles in the elliptic areas with dashed lines. (F) Avidin^SR^fluorescent signal from EYFP^+^MCs in PBS- or BV-serum sensitized ears 30 min after BV injection shown as mean fluorescence intensity (MFI, upper panel) and mean integrated intensity (MII, lower panel). (A-C, E and F) data are from one of two independent experiments, each of which gave similar results). (D) results are pooled from two independent experiments with 9–20 mice per group; symbols represent mean +/− SEM(C and D) Two-way ANOVA with Tukey’s test for multiple comparisons; *p* values are adjusted for multiple testing; *p ≤ .05; **p ≤ .01; ***p ≤ .001

We next used *in vitro* and *in vivo* imaging approaches to characterize the effects of IgE sensitization on MC degranulation at the single-cell level. Stimulation with BV induced a strong and rapid increase in avidin^SR^ (sulforhodamine 101-tagged avidin) signal in individual FSCMCs sensitized with BV serum (Figure 2A–C and Videos S1 and S2). Anti-IgE pre-treatment abrogated this response down to levels comparable to PBS serum-sensitized cells (Figure 2A–C and Video S3), confirming the essential role of IgE in the FSCMC venom response.

To investigate the *in vivo* relevance of IgE-enhanced MC responsiveness to BV, we injected mice intradermally with either BV serum or PBS serum. Subsequent intradermal BV challenge of PBS serum-mock-sensitized ears led to sustained ear swelling for at least3 h (Figure 2D). Interestingly, BV serum significantly increased the immediate tissue response detected 30 min after venom injection. Based on the *in vitro* observations described above, we suspected that the increased swelling induced by BV at sites injected with BV serum might be related to increased MC degranulation. We therefore used *Mcpt5-Cre; R26YeYFP* reporter mice (which express eYFP specifically in mMCP5^+^ connective tissue MCs) and intradermally injected avidin^SR^ to specifically label MC granules.^23^ One week later, the mice were injected in the ear with either PBS serum or BV serum. Intravital 2 photon microscopy performed immediately after BV challenge on the next day revealed increased skin MC degranulation in BV serum-sensitized ears (Figure 2E,F).

Taken together, our results indicate that anti-B V IgE has significant potential for increasing the MC’s response to BV *in vitro* and *in vivo*.

### 3.2 | BV and IgE-mediated signals together enhance FSCMC activation

**FIGURE 3:**
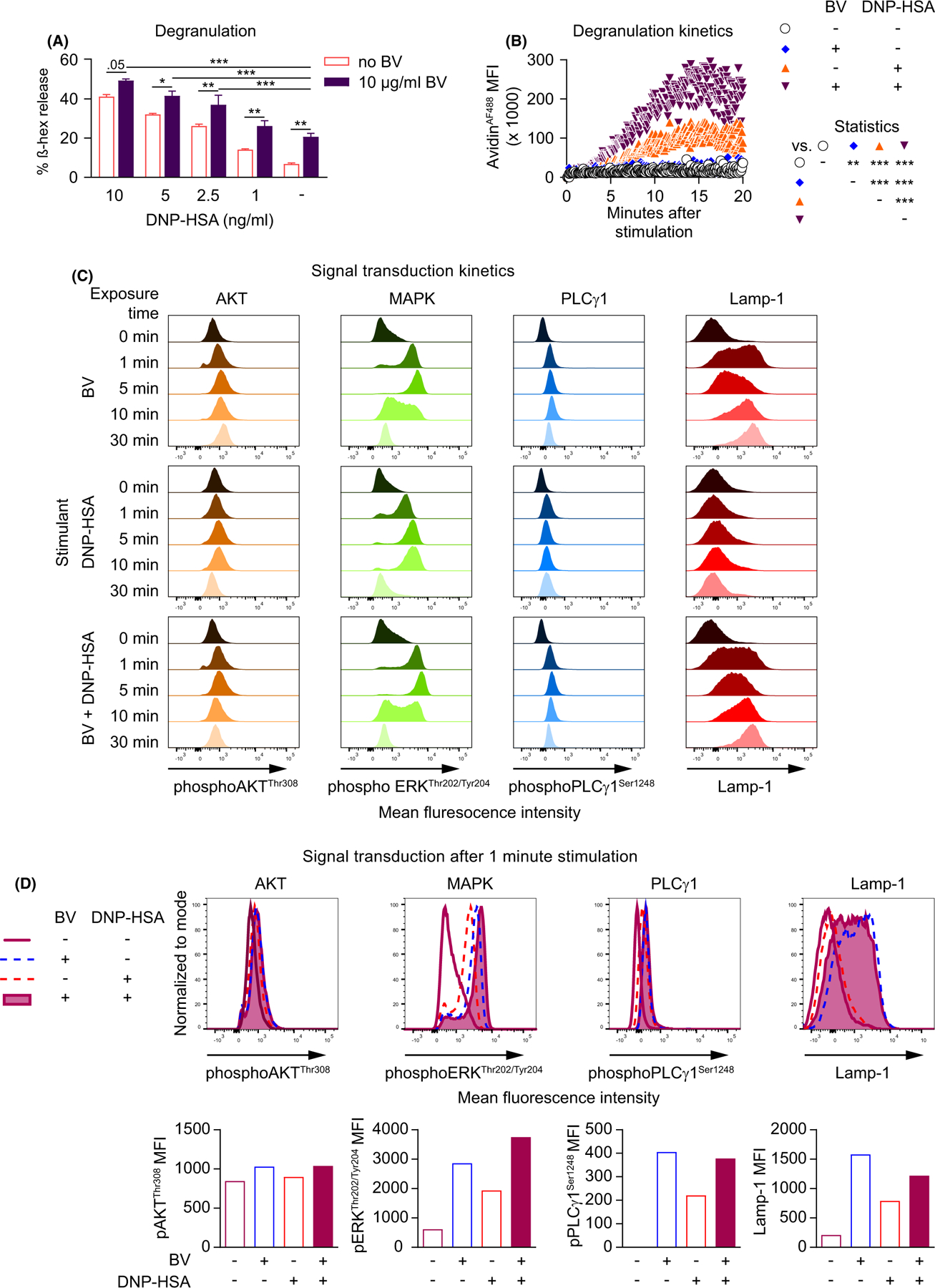
Immunoglobulin E-dependent and BV-mediated signals together increase FSCMC activation. (A–D) FSCMCs were sensitized overnight with anti-DNP IgE. (A) FSCMCs were unstimulated or stimulated with different doses of antigen (DNP-HSA) in the absence or presence of 10 µg/ml BV. Released ß hexosaminidase (ß-hex; % of total) was determined 1 h after stimulation. (B and C) FSCMCs were either not stimulated or treated with either 10 µg/ml BV or 5 ng/ml DNP-HSA or both simultaneously. (B) Realtime fluorescent signal due to binding of avidin^AF488^of live cells over 20 min recorded by flow cytometry. The avidin^AF488^ mean fluorescence intensity (MFI) kinetics are shown. (C) Phosphorylation of AKT, MAPK/ERK, and PLCγ1 and membrane-localized Lamp-1 before (0 min) and after stimulation for 1, 5, 10, or 30 min using (phosphor-) flow cytometry. (D) Selected histogram overlays (upper panels) and respective mean fluorescence intensities (MFIs; lower panels) of phosphorylation data 1 min after stimulation as shown in (C). (A–D) data are from one of at least two independent experiments, each of which gave similar results. (A) graphs represent mean +SD;(A and B) Two-way ANOVA with Tukey’s test for multiple comparisons; *p* values are adjusted for multiple testing;*p ≤ .05; ** *p* ≤ .01; ***p ≤ .001; numbers in (A) show the actual *p* value

We next investigated whether stimulation with BV and IgE directed against a distinct antigen also could enhance MC activation. To do this, we stimulated anti-dinitrophenyl (DNP) IgE-sensitized FSCMCs with different doses of antigen (DNP-coupled to human serum albumin [DNP-HSA]) in combination with a sub-cytotoxic dose of BV (10 µg/ml). We observed that at each tested dose of DNP-HSA, BV significantly increased the release of ß hexosaminidase (Figure 3A). Similarly, BV exposure increased the real-time degranulation kinetics of IgE-sensitized FSCMCs stimulated with 5 ng/ml DNP-HSA (Figure 3B). At this dose of DNP-HSA, BV stimulation exhibited AKT, ERK, and PLCγ phosphorylation (Figure 3C) and a greater effect on ERK phosphorylation than stimulation with BV alone at 1 min (Figure 3D) and 5 min (Figure S2A) after exposure. We conclude from these results that simultaneous stimulation with BV and IgE and antigen can induce a higher activation response in mast cells than either of the individual stimuli on its own.

### 3.3 | IgE-mediated activation increases the detoxification potential of mouse and human MCs

Having observed the effects of IgE (directed against either BV or DNP-HSA) sensitization on MC responses to BV, we next wanted to address the potential biologic relevance of this phenomenon. Higginbotham and Karnella^26^ suggested that MCs might play an important role in host defense against BV-induced cytotoxicity, and this later was proven by tests employing MC-deficient mice.^14^ However, the exact mechanisms by which MCs mediated detoxification of BV were unclear. Phospholipase A_2_(PLA_2_) is an important enzyme found in a wide variety of venoms.^7^ Together with the cytolytic agent melittin, PLA_2_ is considered a major BV constituent (approximately 50% and 12% of dry BV, respectively) and both compounds contribute to the diverse toxic effects of the venom.^13^ Accordingly, either PLA_2_ or melittin might be potential targets of MC-related detoxification mechanisms.

**FIGURE 4:**
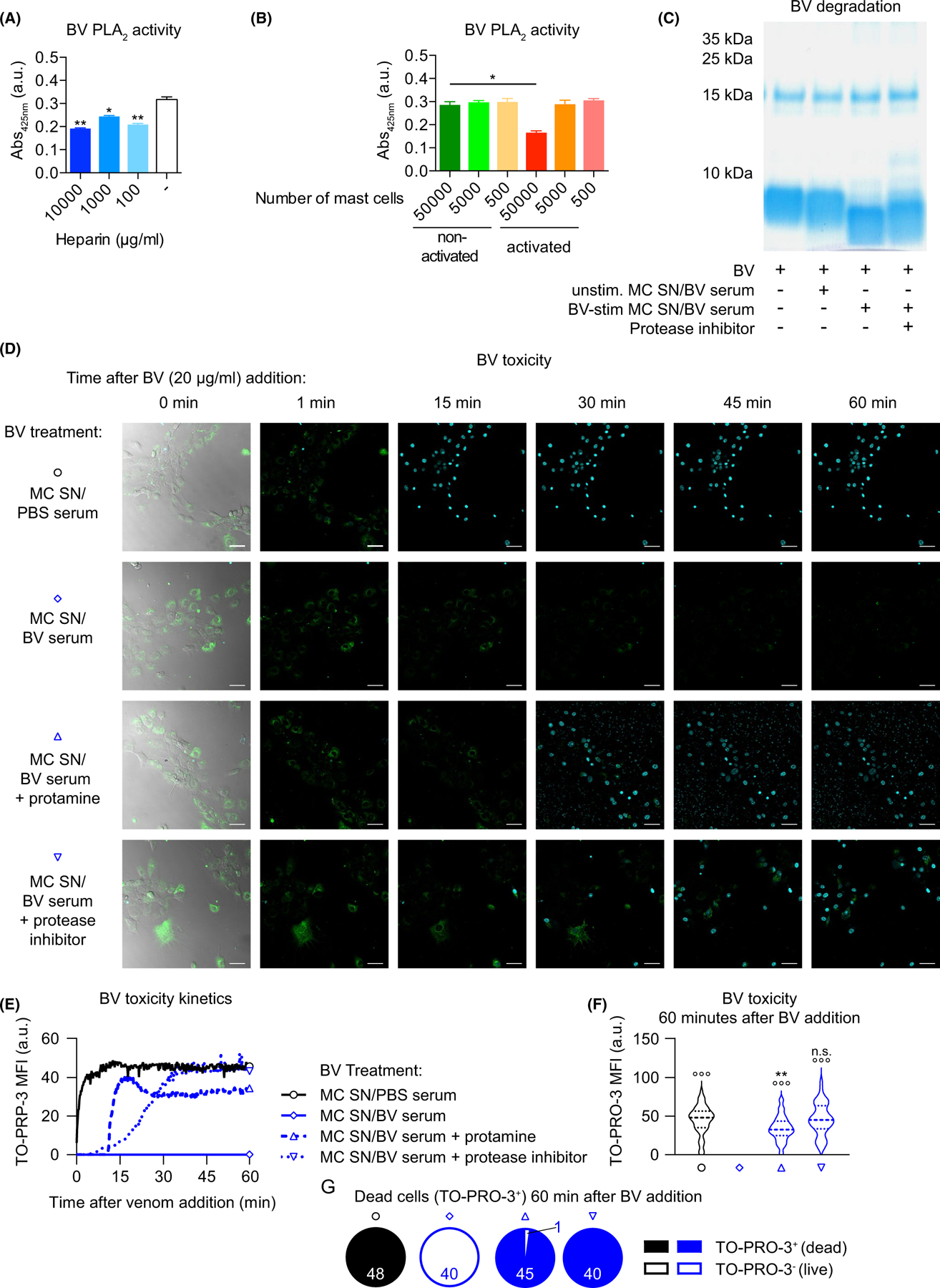
Immunoglobulin E-mediated activation increases the detoxification potential of mouse FSCMCs. (A and B) BV (12.5 µg at 500 µg/ml final concentration, resulting in a PLA_2_ concentration of approximately 60 µg/ml) was either mixed with (A) indicated amounts of heparin or buffer or (B) supernatant of DNP-HSA-exposed non-sensitized or anti-DNP IgE-sensitized MCs for 10 min. After mixing with PLA_2_ substrate and incubation, substrate cleavage (as indicator of PLA_2_ activity) was measured. (C) BV (10 µg) was incubated (or not) with supernatant (MC SN) of unstimulated (unstim.) or BV-exposed (10 µg/ml; for 1 h) BV serum-sensitized FSCMCs for 1 h. In some cases, supernatant of activated FSCMCs was pre-incubated with protease inhibitor. After incubation of BV and supernatants, the mixtures were separated by PAGE, followed by Coomassie blue stain. (D–G) 3 T3 fibroblasts were seeded in chamber slides and stained with Fluo-4 (green). BV was pre-treated (or not) with protamine (100 µg)- or protease inhibitor (1x final concentration)-treatedMC SN of PBS serum or BV serum-sensitized FSMCs that were exposed to 10 µg/ml BV for 1 h. The pre-treated BV was then transferred onto the fibroblasts and monitoring of cell death, by confocal fluorescence microscopy imaging of TO-PRO-3 (turquoise)-positive nuclei over 60 min, was started immediately. (D) Representative pictures (outermost left: merge of brightfield, Fluo-4 and TO-PRO-3 fluorescence; remaining pictures show merges of Fluo-4 and TO-PRO-3 channels) of the field of views at indicated timepoints after addition of MC SN-treated BV are shown. Turquoise stains are nuclei of dead cells. The scale bars represent 10 µm. (E–G) Quantification of toxicity (measured as TO-PRO 3 signal) kinetics from time lapse microscopy data shown in (D). (E) shows the development of TO-PRO 3 mean fluorescence intensity (MFI) over time. (F) Depicts the TO-PRO-3 MFI 60 min after BV addition in all cells in the field of view. (G)Illustrates the proportions of dead (with MFI ≥1) and live (with MFI <1) cells (numbers indicate the respective identified and quantified nuclei) in the field of view. (A–G) data are from one of at least two independent experiments, each of which gave similar results. (A and B) *T* test for pairwise comparisons in (A) to the control group (no heparin) or (B) as indicated; graphs represent mean +SD; (F) one-way ANOVA with Tukey’s test for multiple comparisons (*p* values are adjusted for multiple testing); * (or n.s.—not significant) indicates comparisons to the respective untreated (or PBS serum) group (or as indicated); ° indicates comparisons with the BV serum group; *p* values are adjusted for multiple testing; */°*p* ≤ .05; **/°°*p* ≤ .01; ***/°°°*p* ≤ .001

Using a chromogenic assay, we found that heparin, an abundant glycosaminoglycan with scaffolding functions in MC granules,^2^ can efficiently interfere with the phospholipase activity of BV (Figure 4A; of note, the PLA_2_ concentration in this assay with complete BV should be approximately 60 µg/ml). Similarly, anti-DNP IgE-sensitized MCs that were activated by DNP-HSA for 1 h profoundly decreased BV phospholipase activity (Figure 4B). In addition to heparin, activated MCs also rapidly release an array of MC-specific and non-specific proteases (such as carboxypeptidase A3 [CPA3], a chymase[mMCP4], tryptases, and other serine peptidases). Some of these have been shown to provide benefit by mediating the degradation of endogenous toxic substances, such asendothelin^15,27^ or vasoactive intestinal peptide,^16^ or exogenous compounds contained in scorpion^16^ or reptile^14–16^ venoms.

We performed PAGE (polyacrylamide gel electrophoresis) to assess the potential proteolytic effects of FSCMC compounds on the BV proteome. Exposure of BV to supernatants of IgE-activated FSCMCs resulted in a shift in the low molecular weight venom fraction (below 10 kDa), presumably caused by degradation of toxins into smaller fragments (Figure 4C). This effect could be partially rescued by pre-treatment of the FSCMC supernatant with a broad-spectrum protease inhibitor (Figure 4C). The predominant peptide in this low molecular weight BV fraction that was visibly modified by MC proteases is melittin (which has a molecular weight of approximately 2.8 kDa; Figure S3A), the main BV toxin with high cytolytic activity.^13^

To assess the effects of the FSCMC supernatant on BV cytotoxicity, we exposed mouse 3 T3 fibroblasts to BV and monitored the cell death kinetics over 1 h using real-time confocal fluorescence microscopy. The venom exhibited high cytotoxic potency, with a substantial proportion of the cells dying or dead (ie, viability dye-positive) within a few minutes after starting the recording (Figure 4D–G and Video S4). Remarkably, pre-exposure of BV with supernatant of IgE/antigen-activated FSCMCs almost completely abrogated its cytotoxicity (Figure 4D–G and Video S5).

To test the contributions of released heparin and proteases to the observed BV detoxification, we pre-treated FSCMC supernatant with protamine, a heparin-antagonist,^28^ or with a cocktail of protease inhibitors which do not mediate cytotoxicity (Figure S4A,B). Interestingly, while either treatment delayed the kinetics of BV-induced cytotoxicity, the fibroblasts eventually died. This suggests that both heparin- and protease-mediated mechanisms can contribute to efficient BV detoxification in this model (Figure 4D–G and Videos S6 and S7).

**FIGURE 5:**
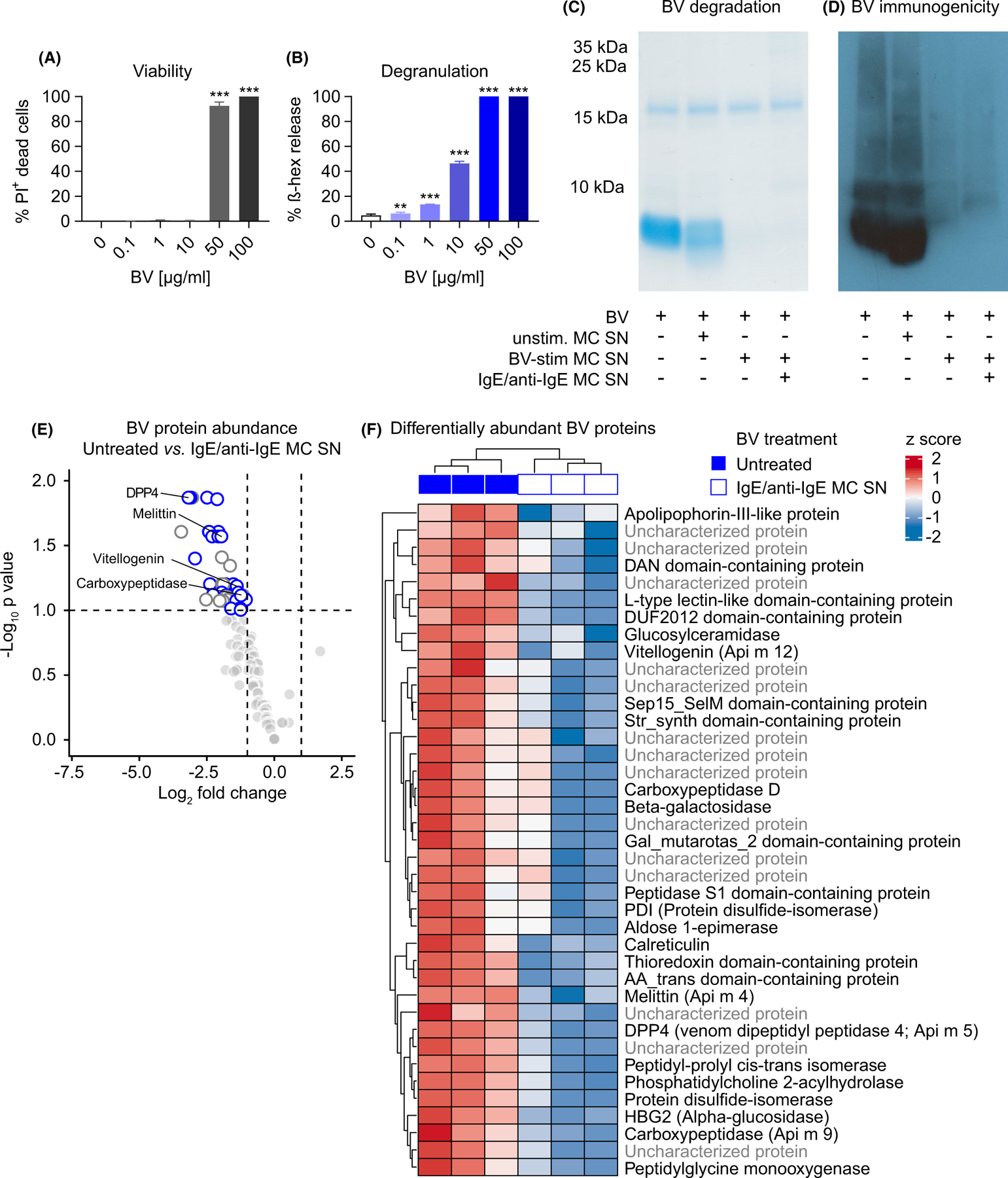
Immunoglobulin E-activated human PBCMCs efficiently degrade BV toxins. (A –B) Human peripheral blood-derived cultured MCs (hu PBCMCs) were stimulated with the indicated concentrations of BV and analyzed after 1 h. (A) Viability assessed by flow cytometry as percentage of PI^+^ among all c-Kit^+^ FcεRIα^+^cells. (B) ß hexosaminidase (ß-hex) released into the supernatant (% of total ß-hex-mediated signal). (C and D) BV (10 µg) was exposed to supernatant (MC SN) collected from either unstimulated (unstim.), 10 µg/ml BV-stimulated (BV-stim), or IgE/anti-IgE stimulated (for 1 h) hu PBCMCs and then processed by PAGE. (C) Coomassie blue-stained gel. (D) Western blot showing signals of IgG antibodies after incubation with BV serum. (E and F) Mass spectrometry analysis (of sample triplicates) of BV after 1 h exposure to supernatant of IgE/anti-IgE-stimulated hu PBCMCs (vs. untreated BV). In total, 118BV proteins were identified. (E) Volcano plot or (F) heatmap depicting the abundance of identified BV proteins. (E) BV proteins with significantly different abundance are highlighted (see panel F or Table S1 for full designations). Of the 39 proteins with lower abundance (FDR 0.1), the characterized compounds are indicated as white circles with blue border, the uncharacterized ones are indicated as white circles with gray border. Known allergens are annotated. (F) Heatmap of BV protein abundance of compounds with a statistically significantly different abundance (FDR 0.1). Raw and adjusted *p* values and fold changes are shown in Table S1. Raw abundances were log_2_ and z-score transformed prior to visualization. (A–D) data are from one of at least two independent experiments each of which gave similar results. (A and B) graphs represent mean +SD; one-way ANOVA with Dunnett’s test for multiple comparisons (*p* values are adjusted for multiplicity analysis); * indicates comparisons with the untreated (0 µg/ml BV) group; *p* values are adjusted for multiple testing; **p ≤ .01; ***p ≤ .001(E and F) Mass spec analysis of sample triplicates was performed once

**FIGURE 6:**
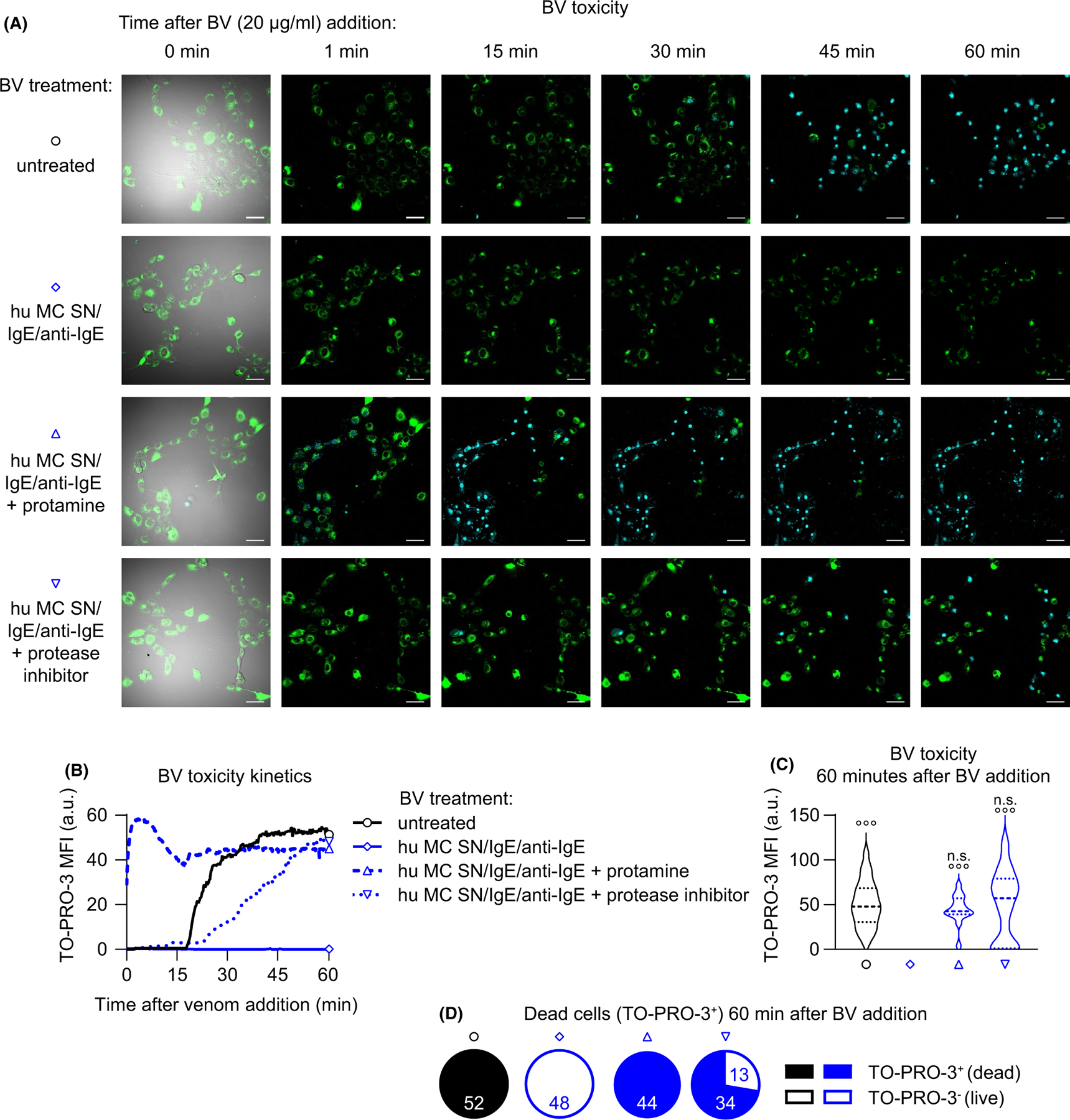
Heparin and proteases released by IgE-activated human PBCMCs interfere with BV toxicity. (A) 3 T3 fibroblasts were seeded in chamber slides and stained with Fluo-4 (green). Untreated BV (20 µg/ml final concentration) or BV pre-treated with supernatants of hu PBCMCs (stimulated for 1 h by IgE/anti-IgE; hu MC SN) that were either untreated or pre-treated with protamine (100 µg) or protease inhibitor (1x final concentration) were transferred onto the fibroblasts, and monitoring by confocal fluorescence microscopy imaging over 60 min was started immediately. Representative pictures (outermost left: merge of bright field, Fluo-4 and TO-PRO-3; remaining pictures show merges of Fluo-4 and TO-PRO-3 channels) of the field of views at different timepoints after venom addition are shown. Turquoise stains are (TO-PRO-3^+^) nuclei of dead cells. (B–D) Quantification of toxicity (measured as TO-PRO-3 signal) kinetics from time lapse microscopy data shown in (A). (B) shows the development of TO-PRO-3 mean fluorescence intensity (MFI) over time. (C) depicts the TO-PRO-3 signal 60 min after BV addition and show (upper panel) MFI all cells and (D) shows the portions of dead (with MFI ≥1) and live (with MFI <1) cells (numbers indicate the respective identified and quantified nuclei per field of view). (A–D) data are representative of at least two independent experiments. (C) One-way ANOVA with Tukey’s test for multiple comparisons (*p* values are adjusted for multiple testing); n.s. (not significant) indicates comparisons to the respective untreated (or PBS serum) group (or as indicated); ° indicates comparisons with the BV serum group; *p* values are adjusted for multiple testing; °°°*p* ≤ .001

We next addressed translational aspects of our findings, using human peripheral blood-derived cultured MCs (hu PBCMCs). Like FSCMCs, hu PBCMCs also exhibited enhanced degranulation when challenged with 1 or 10 µg/ml BV (reflected by increased supernatant ß-hexosaminidase and tryptase activity as well as avidin^AF488^ binding) without detectable cytotoxicity (Figure 5A–B and Figure S5A–B). We then used PAGE to assess the potential of hu PBCMC-released mediators to degrade BV and observed a profound effect of supernatants from hu PBCMCs stimulated for 1 h with either BV (10 µg/ml) or IgE and anti-IgE (Figure 5C and Figure S5C). Importantly, such treatment substantially decreased the immuno-recognition of BV, reflected by a strongly diminished signal mediated by BV-specific serum IgG antibodies in Western blots (Figure 5D). Mass spectrometry analysis revealed that treatment of BV with supernatant collected from IgE-activated hu PBCMCs significantly decreased the abundance of BV toxins, including the major allergens^29,30^ melittin (also known as Api m 4) and venom dipeptidyl peptidase 4 (DPP4, also known as Api m 5), as well as carboxypeptidase (also known as Api m 9) and vitellogenin (also known as Api m 12) (Figure 5E,F; Table S1). Of note, while not reaching statistical significance, many other BV components, such as Icarapin-like (also known as Api m 10) and venom carboxylesterase-6 (also known as Api m 8), also showed lower abundance after supernatant exposure (Figure S5D; Table S1). Finally, detoxification experiments with supernatant collected from IgE and anti-IgE activated hu PBCMCs recapitulated our observations with FSCMCs: hu PBCMC supernatant counteracted BV cytotoxicity in a predominantly heparin but also partially protease-dependent manner (Figure 6A–D and Videos S8–S11).

We conclude from this series of experiments that IgE-activated human PBCMCs and mouse FSCMCs share the potential of detoxifying BV, and that this reflects, at least in part, their release of both heparin and proteases.

## 4 | DISCUSSION

MCs are found in most vascularized tissues of mammals, as well as in fish, reptiles, and urochordates, but their biological function has remained unclear.^4^ As one of just a few cell types, MCs express the high-affinity IgE receptor, FcεRI, in a hetero-tetrameric configuration consisting of one IgE-binding α-chain, one transmembraneß-chain, and two signal-transducing γ-chains (the γ-chain is also shared with several IgG Fc receptors).^31^ This FcεRI configuration allows antigen-specific priming of MCs, with dramatic cellular responses upon antigen exposure.^1^

As this mechanism is central for allergies (with IgEs directed against seemingly innocuous environmental compounds), the physiologic functions of IgEs and MCs have been an important focus of allergy research for decades. One well-accepted function of this *allergy module* (IgEs and MCs) of immunity has been host defense against parasites.^4,6,32^ Indeed, many parasites invade host tissues throughout their life cycle, a process often associated with severe tissue damage, which is considered a major signal initiating (T helper cell) type 2 responses.^33^ Type 2 immune responses and the production of associated cytokines like IL-4 and IL-13 are considered the pre-requisite for the process of antibody class switch required for IgE production by B cells.^34^ While this adaptive immune response can be beneficial and is required for clearing of certain parasite infections,^33,35^ the experimental evidence regarding IgE and MC functions in such settings is controversial.^5^

Initial evidence for an alternative function of MCs in host defense against bee venom has been provided by R. D. Higginbotham and colleagues.^26^ They reported that BV injection induced MC degranulation and that heparin treatment of BV reduced the lethality of the venom upon its intravenous application in mice.^26^ More than 30 years later, our laboratory confirmed that MC-deficient mice exhibited increased susceptibility to subcutaneous BV injections.^14,18^ In subsequent studies, we found that honeybee venom and Russell’s viper venom are potent inducers of type 2 immune responses and IgE production^17,18^ in mice. While venom-specific IgE has been commonly associated with the risk of allergy and anaphylaxis, the alternative toxin hypothesis (originally postulated by Margie Profet) suggested a beneficial function of IgEs in host defense against noxious substances, for instance by increasing the innate detoxifying potential of MCs.^8,9^ The results of our laboratory^17,18^ and the Medzhitov^19^ laboratory provided evidence for this idea; however, the specific molecular consequences of IgE sensitization on MC responses to venoms have not been fully addressed.

In the current study, we found that while certain concentrations of BV can trigger active degranulation (independent of cell lysis) of non-sensitized MCs, sensitization with serum from BV-immunized mice strongly enhanced the MC response in an IgE-dependent manner. The presence of anti-BV IgE increased BV-induced MC granule release on a population and single-cell level *in vitro* and *in vivo*. Furthermore, the MAP kinase-related ERK signaling kinetics were profoundly extended, and gene expression for diverse cytokines and chemokines, were increased in an IgE-dependent manner. These findings indicated that venom-specific IgE can efficiently increase the magnitude of BV-induced MC responses, including the release of preformed and *de novo* produced compounds.

While it has been previously reported that MC CPA3 and mMCP4 can contribute to the degradation of snake venom sarafotoxin^14,15^ and the Gila monster venom constituent helodermin,^16^ the molecular mechanisms of BV detoxification by MC compounds were unclear.^14^ Our data show that heparin and other MC compounds can interfere with the venom’s phospholipase activity, which is thought to mediate cytotoxicity via production of lysophospholipids.^13^ In addition, we observed degradation of several important venom allergens by preformed proteases released by IgE/antigen-activated MCs, including melittin (Api m 4), Api m 5, Api m 9, and Api m 12. However, treatment of MC supernatants with a pan protease inhibitor seemed to not fully restore the PAGE migration pattern of untreated BV, possibly indicating incomplete interference with protease activity. Also, the partial BV digestion due to limited protease inhibition was not sufficient to fully detoxify the venom. In summary, our findings suggest that both heparin and proteases can have important roles in BV detoxification.

In humans, population studies show that healthy individuals (ie, without diagnosed atopic disease) can express IgE specific for hymenoptera venom toxins.^36,37^ This may indicate that toxin-specific IgE hassome non-pathologic function(s) in such people. While occasional bee stings are common in the general population,^11^ beekeepers represent a subpopulation that is regularly exposed to BV. Up to ~30% of beekeepers respond with large local or systemic reactions to bee stings and up to 60% exhibit positive skin tests and detectable BV-specific IgE.^38^ Remarkably, systemic allergic reactions seem to be more frequent in beekeepers who are infrequently stung.^38^ This might be due to the higher levels of venom-specific IgG in such frequently exposed individuals, which outcompetes MC-bound IgE with the same epitope specificity. However, the precise function of BV-specific IgE in the increased venom tolerance of beekeepers^38^ is still not fully understood.

Overall, our study has identified the contribution of two classes of preformed MC compounds, the proteoglycan heparin, as well as proteases, to resistance against BV toxicity. Importantly, we also show that specific serum IgE can substantially increase expression of the detoxifying potential of MCs. These findings thus reveal the significant benefit of this specialized anti-venom module of the adaptive immune system.

## Supplementary Material

sm1

sm2

sm4

sm6

sm7

sm3

sm9

sm8

sm10

sm11

sm5

sm14

sm15

sm12

sm13

sm18

sm16

sm17
