# Supplementary material for: IgE antibodies increase honeybee venom responsiveness and detoxification efficiency of mast cells
Source: Allergy. Author manuscript; Available in PMC 2023 Feb 1. (PMC8502784; doi:10.1111/all.14852)
Supplement: sm2 [file NIHMS1707162-supplement-sm2.docx]

# Appendix S1

# IgE antibodies increase honeybee venom responsiveness and detoxification efficiency of mast cells

## Authors:

Philipp Starkl^1,2,3,^*, Nicolas Gaudenzio^3,4^, Thomas Marichal^3,5^, Laurent L. Reber^3,4^, Riccardo Sibilano^3,6^, Martin L. Watzenboeck^7^, Frédéric Fontaine^2^, André C. Mueller^2^, Mindy Tsai^3,6^, Sylvia Knapp^1,2^, Stephen J. Galli^3,6,8,^*

## Affiliations:

^1^Laboratory of Infection Biology, Dept. of Medicine I, Medical University of Vienna, 1090 Vienna, Austria

^2^CeMM - Research Center for Molecular Medicine of the Austrian Academy of Sciences, 1090 Vienna, Austria

^3^Dept. of Pathology, Stanford University School of Medicine, Stanford, CA, 94305-5176, USA

^4^Toulouse Institute for Infectious and Inflammatory Diseases, INSERM UMR1291, CNRS UMR5051, University of Toulouse III, Toulouse, France.

^5^GIGA-Research and Faculty of Veterinary Medicine, University of Liege, 4000 Liege, Belgium

^6^Sean N. Parker Center for Allergy and Asthma Research, Stanford University, Stanford, CA, 94305-5176, USA

^7^Department of Biomedical Imaging and Image-guided Therapy, Medical University of Vienna, 1090 Vienna, Austria

^8^Dept. of Microbiology and Immunology, Stanford University School of Medicine, Stanford, CA, 94305-5176, USA

*Correspondence: [philipp.starkl@meduniwien.ac.at](mailto:philipp.starkl@meduniwien.ac.at) (P.S.); [sgalli@stanford.edu](mailto:sgalli@stanford.edu) (S.J.G.);

## Supplementary Methods

### Mice

Animal care and experiments were performed following current guidelines of the National Institutes of Health and with approval of the Stanford University Institutional Animal Care and Use Committee (IACUC protocol #12683). Female 6-8 week old mice were used for *in vivo* experiments. C57BL/6 wildtype (wt) mice were obtained from Jackson Laboratories and housed in the Stanford University animal facility for at least 7 days before starting experiments. *Mcpt5-Cre^+^* (*Tg^(Cma1-cre)ARoer^*) mice^1^ were generously provided by Axel Roers (University of Technology, Dresden, Germany). *R26Y EYFP* (B6.129X1-*Gt(ROSA)26Sor^tm1(EYFP)Cos^*/J) mice^2^ were originally obtained from Jackson Labs.

### Reagents and antibodies

Honeybee venom (all of Lot 12071006HB) was obtained from ALK Abello Source Material. The freeze-dried complete BV was resuspended in sterile, endotoxin-free PBS (Gibco) at 4 mg/ml and stored at -20°C. Mouse anti-dinitrophenyl (DNP) IgE (clone e26) was kindly provided by Fu-Tong Liu^3^ (then at University of California-Davis). Purified bee venom phospholipase A_2_ (PLA_2_), purified bee venom melittin, Dinitrophenyl_30-40_-conjugated human serum albumin (DNP-HSA), heparan sulfate (heparin), p-nitrophenyl-N-acetyl-ß-D-glucosaminide (pNAG), supersensitive TMB substrate, dimethyl sulfoxide (DMSO), DNAse I, avidin-sulforhodamine 101 (avidin^SR^) and protamine sulfate (protamine) were obtained from Sigma. EZ-Link Sulfo-NHS-SS-Biotin, 3.5K molecular weight cutoff Slide-A-Lyzer Cassettes and 2K molecular weight cutoff Zeba Spin Desalting Columns were obtained from Thermo Fisher Scientific. AlexaFluor^488^-labelled avidin (avidin^AF488^) was obtained from Invitrogen. Protease activity was inhibited using the Halt Protease Inhibitor Cocktail (Thermo Fisher Scientific). PLA_2_ activity was assessed using 4-nitro-3-(octanoyloxy)-benzoic acid (4N3OBA; Enzo Life Sciences). Rat anti-mouse IgE (clone R35-92; for blocking of IgE binding to FcεRIα) and the respective rat IgG1 isotype control (clone R3-34) were obtained from BD Pharmingen. Human myeloma IgE was from Calbiochem, polyclonal rabbit anti-human IgE were obtained from Bethyl Laboratories. Anti-human FcεRIα (Clone AER-37/CRA1) was obtained from eBioscience, anti-human CD117 (c-Kit; clone 104D2) and anti-human tryptase (clone AA1) were obtained from BioLegend. Anti-mouse FcεRIα (clone Mar-1) and anti-mouse CD117 (c-Kit; clone 2B8) were obtained from BioLegend. Anti-Phospho-Akt (Thr308; clone D25E6), anti-Phospho-p44/42 MAPK (Erk1/2) (Thr202/Tyr204; clone 197G2), anti-Phospho-PLCγ1 (Ser1248; clone D25A9) (all from Cell Signaling Technology) were used for phospho-flow cytometry analysis. Serum ELISA reagents rat anti-mouse IgE (clone R35-72), biotinylated rat anti-mouse IgE (clone R35-118), purified mouse IgE (clone C38-2), biotinylated rat anti-mouse IgG1 (clone A85-1), biotinylated rat anti-mouse IgG2b (clone R12-3) and horseradish peroxidase-conjugated streptavidin were all from BD Pharmingen, polyclonal goat anti-mouse IgG2c was obtained from Southern Biotech. Goat anti-mouse IgG (H+L) Cross-adsorbed horse radish peroxidase-labelled HRP antibody was from Thermo Fisher Scientific.

### Serum generation, characterization and processing

For generation of mouse sera, C57BL/6 wt mice were shaved on the back skin and received two subcutaneous injections of 50 µl (containing 200 µg) BV or PBS in the shaved area. Three weeks later, mice were sacrificed and blood was collected by cardiac puncture, followed by centrifugation in Serum-Gel Microtubes (Sarstedt), pooling of the sera, and freezing and storage at -20°C.

Mouse sera (either of aliquots from individual animals collected before pooling or from serum pools) were analyzed by ELISA as previously described^4,5^. Briefly, MaxiSorp ELISA plates (Nunc) were coated with 50 µl BV (5 µg/ml in PBS) or anti-mouse IgE (2 µg/ml in PBS) per well overnight at 4°C. The next day, the plate was washed 3-5 times using PBS containing 0.05% tween (as was done after each subsequent incubation step) and unspecific binding sites were blocked by incubation with 200 µl of 1% bovine serum albumin (BSA) in PBS for at least 2 hours at room temperature. Fifty µl serial-diluted individual sera (in PBS containing 1% BSA) and reference immune serum (pool) or IgE isotype (as standard) were added to the blocked wells and incubated 2 h at 37°C. Next, 50 µl biotinylated detection antibodies specific for mouse IgG1, IgG2b, IgG2c or IgE (all diluted 1:1000 in PBS containing 1% BSA) were added for 1 hour at room temperature, followed by 30 minutes incubation with 50 µl horseradish peroxidase-conjugated streptavidin (diluted 1:2000 in PBS containing 1% BSA) and detection using 50 µl supersensitive TMB substrate (and reaction stop by addition of 50 µl 2 N H_2_SO_4_). Absorbance at 450 nm (with 620 nm as reference) was detected using a Tecan Sunrise plate reader. IgG1, IgG2b and IgG2c titers were determined by plotting the serum dilution that gave half-maximal signal of the reference serum. Absolute IgE levels were calculated based on the IgE isotype standard curve.

We detected BV PLA_2_-specific IgE by ELISA as previously described with minor modifications^5^. Briefly, purified BV PLA_2_ (4 mg/ml PBS) was mixed 1:1 with EZ-Link Sulfo-NHS-SS-Biotin (2 mg/ml in DMSO) and incubated for 2 hours at room temperature. After quenching with 1 volume 0.1 M TRIS (pH 7.0) for 10 minutes, the solution was dialyzed twice overnight against PBS at 4°C using Slide-A-Lyzer Dialysis Cassettes, passed over Zeba Spin Desalting Columns (according to the manufacturer’s instructions) and eluted again in PBS. The ELISA to detect BV PLA_2_-specific IgE was performed as described above for total IgE, replacing the biotinylated anti-IgE antibody with 1:2000-diluted (identified as suitable dilution with lowest signal-to-noise ratio in pilot experiments using mouse immune sera) biotinylated BV PLA_2_ and extending the incubation time in this step to 3 hours at 37°C. The mean of all PBS serum pool signals (Absorbance_450-620nm_) was subtracted as background from all samples.

For neutralization of IgE activity in selected experiments, BV serum was treated for 30 minutes with either 10 µg/ml anti-mouse IgE or with isotype control. PBS serum was treated with isotype control antibody in the respective experiments^4^. Alternatively, IgE was inactivated by heating at 56°C in a water bath for 45 minutes^4^.

### Cellular models

#### Fetal skin-derived cultured mast cells (FSCMCs)

FSCMCs were generated following a published protocol with few modifications^6^. Pregnant C57BL/6 wt mice were euthanized on day 17 or 18 of pregnancy. Fetuses were harvested and rinsed 2 times in sterile endotoxin-free PBS. Whole fetuses were then incubated in 0.05% Trypsin EDTA with phenol red (Gibco, Thermo Fisher Scientific) supplemented with 100 u/ml DNAse I for 30 minutes at 37°C in 12 well plates (1 fetus/well). The trypsin suspensions were then pooled and filtered over a 70 µm cell strainer (BD Biosciences) and RPMI 10% FBS was added to stop digestion. After centrifugation for 5 min at 300 g, cells were resuspended in complete culture medium (RPMI 10% fetal bovine serum [FBS], 10 mM HEPES, 1x penicillin/streptomycin, 1x nonessential amino acids, 1 mM sodium pyruvate, 50 µM ß-mercaptoethanol; all from Gibco apart from FBS which was from Sigma) supplemented with 10 ng/ml IL-3 and 10 ng/ml SCF (both from Peprotech). Cells from 3 fetuses were seeded in 100 ml medium in 175 cm^2^ tissue culture flasks and maintained for 4 weeks without medium change. Then, cells were harvested and seeded in 50 ml fresh medium (including cytokines) for 2-4 additional weeks before assessment of maturation by flow cytometry (more than 95% FcεRIα/c-Kit double positive cells). Cells were used between 6 – 12 weeks after start of the culture.

#### Human peripheral blood mononuclear cell-derived mast cells (PBCMCs)

PBCMCs were generated as previously described^7^. Briefly, CD34^+^ precursor cells were isolated from peripheral blood mononuclear cells derived from buffy coats of healthy donors (Stanford Blood Center) using the EasySep Human CD34 Positive Selection Kit (Stemcell Technologies). After culture for one week in StemSpan medium (Stemcell Technologies) supplemented with human SCF (50 ng/ml), IL-3 (10 ng/ml) and IL-6 (50 ng/ml; all cytokines were from Peprotech), the cells were maintained in IMDM Glutamax I, supplemented with 1 mM sodium pyruvate, 50 mM ß-mercaptoethanol, 0.5% bovine albumin fraction V, insulin-transferrin selenium (all from Invitrogen/Thermo Fisher Scientific), ciprofloxacin (10 µg/ml; Sigma), IL-6 (50 ng/ml) and SCF (50 ng/ml). Cell maturity was tested by flow cytometry staining for tryptase, c-Kit and FcεRIα expression. PBCMCs were typically used for experiments after approximately 10 weeks of culture.

### Gene expression analysis

FSCMCs were seeded at 5x 10^5^ cells/ml and incubated 1:20 with PBS serum or BV serum (either pre-treated with anti-IgE or isotype control antibody or with heating to 56^o^ C as described above). The next day, the cells were washed and stimulated with 10 µg/ml BV (or the respective amount of PBS) in RPMI 2% BSA for 1 hour in 96 well round bottom plates. Cells were centrifuged 5 minutes at 300 g and pellets were resuspended in Trizol (Invitrogen), followed by RNA isolation. One µg of RNA was transcribed into cDNA using the High Capacity cDNA Reverse Transcription Kit (Applied Biosystems). Real-time PCR was conducted on a StepOne Plus device (Applied Biosystems) using Power SYBR reagents (Applied Biosystems). Target gene Ct levels were normalized to the housekeeping gene *eef1b2* and relative gene expression compared to non-stimulated samples was calculated using the 2^-ΔΔCt^ algorithm. Primer sequences:

*eef1b2*: fwd: AGAGCTACATTGAGGGGTACGT; rev: GACTTGATGTGATTATACCAACGTAG;

*il1b*: fwd: GCTTCCTTGTGCAAGTGTCTGAA rev: GAACAGGTCATTCTCATCACTGTCA;

*il4*: fwd: GTCCTCACAGCAACGAAGAACACCA; rev: CTCATTCATGGTGCAGCTTATCGA;

*il5*: fwd: TGTTGACAAGCAATGAGACGATGA; rev: GGACAGTTTGATTCTTCAGTATGTC;

*il6*: fwd: GAAGTTCCTCTCTGCAAGAGAC; rev: GTATCCTCTGTGAAGTCTCCTCT;

*il10*: fwd: CTTATCGGAAATGATCCAGTTTTACC; rev: ATCACTCTTCACCTGCTCCACTG;

*il13*: fwd: TATTGAGGAGCTGAGCAACATCAC; rev: TCTGGGTCCTGTAGATGGCA;

*il18*: fwd: ACACGCTTTACTTTATACCTGAAG; rev: TACTGCGGTTGTACAGTGAAGTC;

*tnf*: fwd: CTCCCTCTCATCAGTTCTATGG; rev: CTCCACTTGGTGGTTTGCTAC;

*mip1a*/*ccl3*: fwd: CTCTGTACCATGACACTCTGCA; rev: CTCTTAGTCAGGAAAATGACACCTG;

*mip2*/*cxcl2*: fwd: GTGAACTGCGCTGTCAATGCCTGA; rev: CTTGAGAGTGGCTATGACTTCTGTCT;

*gm-csf*/*csf2*: fwd: GATGACATGCCTGTCACGTTGAAT; rev: GTCTGCACACATGTTAGCTTCTTGA;

*ccl1*: fwd: TCTCCAATAGCTGCTGCTTGAACA; rev: ACCTTTGTTCAGCCTGAATACCACA;

*ccl2/mcp1*: fwd: GTGTTGGCTCAGCCAGATGC; rev: ACCTCTCTCTTGAGCTTGGTGACA;

*ccl4*: fwd: GTTCTCAGCACCAATGGGCTCTGA; rev: AGCAAAGACTGCTGGTCTCATAGTA;

*ccl8*: fwd: GAAGCTGTGGTTTTCCAGACCA; rev: CTGGTCAAGGATCTCCATGTAC;

### Signal transduction analysis by phospho-flow cytometry

FSCMCs were seeded at 5x 10^5^/ml in RPMI medium supplemented with 10% FBS and 1x penicillin/streptomycin (without cytokines) and incubated overnight with 1:20 PBS serum or BV serum processed as described above. The next day, cells were washed with RPMI medium without additives (plain RPMI), resuspended at 5x 10^6^/ml of pre-warmed plain RPMI and aliquoted at 100 µl into FACS tubes and incubated at 37°C for at least 5 minutes. Cells were then stimulated by quick addition of 100 µl pre-warmed plain RPMI or RPMI containing 20 µg BV/ml and immediately put back to 37°C. At indicated timepoints, 1 ml of 4% paraformaldehyde was added and mixed by pipetting to fix the cells for 15 minutes at room temperature. Fixed cells were then washed with 2 ml ice-cold PBS 1% BSA (FACS buffer) and resuspended in 300 µl ice-cold (by storage at -20°C) methanol and incubated 30 minutes on ice. Next, cells were washed with 2 ml ice-cold FACS buffer and resuspended in 100 µl FACS buffer containing 1:100 phospho-flow and surface stain (anti-c-Kit and anti-FcεRIα) antibodies. After incubation for 2 hours on ice, cells were washed 2x with FACS buffer and analyzed by flow cytometry using an LSR-II flow cytometer (BD Biosciences) at the Stanford University Flow Cytometry Core Facility.

In experiments dissecting the contribution of innate BV stimulation on IgE/antigen (DNP-HSA) mediated signaling, FSCMCs were processed as described above, replacing serum with 1 µg/ml anti-DNP IgE and stimulated with either 5 ng/ml DNP-HSA, 5 µg/ml BV, or simultaneously stimulated with both, DNP-HSA and BV.

### Degranulation assays

For analysis of MC degranulation, 5x 10^5^ FSCMCs/ml were sensitized 1:20 with PBS serum or BV serum (sera processed as described above) overnight. The next day, the cells were centrifuged for 5 minutes at 300 g, washed with Tyrode’s buffer (100 mM HEPES, 1.8 mM CaCl_2_, 5 mM KCl, 130 mM NaCl, 2 mM MgCl_2_, 5.5 mM glucose and 1 g/L BSA; pH 7.4), resuspended at 2x 10^6^ cells/ml in Tyrode’s buffer and aliquoted at 20 µl in 96 well v-bottom plates. Next, 20 µl of 2x stimulants in Tyrode’s buffer were added and cells were incubated for 1 hour at 37°C. The chromogenic substrate pNAG was used to assess the release of ß-hexosaminidase (a granule-stored enzyme) into the supernatant as percentage of the combined signal of cell lysate and supernatant, as previously described^8^.

In experiments dissecting the influence of innate BV stimulation on IgE/antigen (DNP-HSA)-mediated degranulation, FSCMCs were processed as described above, replacing serum with 1 µg/ml anti-DNP IgE and stimulated with either DNP-HSA or BV, or simultaneously stimulated with both DNP-HSA and BV, at the indicated concentrations.

In experiments assessing the degranulation of human MCs, 2x 10^4^ hu PBCMCs were stimulated with different venom dosages as indicated for 1 hour at 37°C. Degranulation was assessed as described above for FSCMCs. For assessment of tryptase activity, cell supernatants and lysates were prepared as described above and 20 µl of each were incubated with 20 µl of the chromogenic substrate S-2288^9^ (3 mM final concentration; Chromogenix) for 1 h at 37°C, followed by detection at 405 nm using a Tecan Sunrise plate reader.

### MC viability upon BV exposure

FSCMCs and human PBCMCs (10^5^ cells/sample) were incubated in Tyrode’s buffer with BV at the indicated concentrations for 1 hour at 37°C. Subsequently, cells were centrifuged 5 minutes at 300 g and washed with FACS buffer (see above) and stained for 20 minutes on ice with antibodies for FcεRIα and c-Kit diluted 1:100 in FACS buffer. After additional washing, cells were resuspended in FACS buffer containing 1:500 propidium iodide (Invitrogen) and analyzed by flow cytometry.

### Degradation of BV

For BV digest experiments with FSCMC supernatants, cells were sensitized overnight with PBS or BV serum. The next day, cells were washed and resuspended in Tyrode’s buffer at 8x 10^6^ cells/ml. Cells were then incubated in 1.5 ml microcentrifuge tubes by addition of an equal volume of 20 µg/ml BV in Tyrode’s buffer for 1 hour at 37°C. For digest experiments with hu PBCMC supernatants, cells were sensitized at 5x 10^5^ cells/ml in culture medium overnight with 2 µg/ml human myeloma IgE. The next day, hu PBCMCs were washed and resuspended in RPMI without phenol red (and additives; RPMI w/o PR; Gibco) at 10^7^ cells/ml and stimulated with an equal volume of 20 µg/ml BV or 1 µg/ml anti-human IgE for 1 hour at 37°C. After stimulation, cells were resuspended by pipetting and centrifuged 5 minutes at 300 g. Supernatants were collected and stored at -80°C.

For assessment of digestion capacity, supernatants were thawed and 10 µl BV (1 mg/ml) was incubated with 20 µl of MC supernatant in the presence or absence of protease inhibitor (40 µl total reaction volume) for 1 hour at 37°C. Poly-acrylamide gel electrophoresis (PAGE) of samples was performed under reducing conditions using the Biorad Criterion System and 18 % Tris-HCl Precast (Fig. 4C and 5C) or 15 % Tris-HCL self-made (Fig. S3A) Gels. Gels were stained using the PageBlue Protein Staining Solution (Thermo Fisher Scientific).

In experiments titrating the number of hu PBCMCs required for efficient BV digestion, supernatants of IgE/anti-IgE-activated MCs were diluted to contain the mediators secreted by 10^4^, 5x 10^4^ or 10^5^ cells in 20 µl.

For analysis of immunorecognition of BV after treatment with SN of hu PBCMCs, PAGE-separated untreated and treated BV (see above) was transferred onto a PVDF membrane (BioRad) using the Trans-Blot SD Semi-Dry Electrophoretic transfer cell (BioRad; 40 V, 2 hours). After at least 2 hours blocking (all incubation and washing steps were performed under gentle shaking) in 5% BSA in Tris-buffered saline (TBS) at room temperature, the membrane was incubated over night at 4°C with 1:5 BV serum in TBS containing 1% BSA. After washing 3x 10 minutes with TBS containing 0.1 % Tween (Sigma; TBST), the membrane was incubated 1 hour at room temperature with goat anti-mouse IgG (H+L) Cross-adsorbed horse radish peroxidase-labelled HRP antibody (Thermo Fisher Scientific) diluted 1:10000 in TBST containing 1% BSA. After 6x 20 minutes washing in TBST, bound antibody was detected using the SuperSignal West Pico Plus Chemiluminescent Substrate (Thermo Fisher Scientific).

### Mass spectrometry

For mass spectrometry analysis, triplicates of 20 µg BV (5 µl at 4 mg/ml) were mixed with 10 µl of SN of hu PBCMCs stimulated by IgE/anti-IgE for 1 hour prepared as described above in section “BV digest analysis”. After 60 min incubation at 37°C (with controls containing only BV or hu PBCMC supernatant, respectively, processed in parallel), samples were frozen in liquid nitrogen and stored at -80°C until analysis. For mass spectrometry analysis, samples were processed using adapted Single-Pot solid-phase-enhanced sample preparation (SP3) methodology^10^. Briefly, equal volumes (125 μl containing 6250 µg) of two different kind of paramagnetic carboxylate modified particles (SpeedBeads 45152105050250 and 65152105050250; GE Healthcare) were mixed, washed three times with 250 µl water and reconstituted to a final concentration of 50 μg/μl with LC-MS grade water (LiChrosolv; MERCK KgaA). Samples (15 µL) were filled up to 50 µL with LC-MS grade water and mixed with 2x sample buffer (4% SDS, 100mM HEPES, pH 8.0) to a final concentration of 2% SDS, proteins were reduced with a final concentration of 10 mM DTT and incubated at 56°C for 1 hour. After cooling down to room temperature, reduced cysteines were alkylated with iodoacetamide at a final concentration of 55 mM for 30 min in the dark. For tryptic digestion, 400 μg of mixed beads were added to reduced and alkylated samples, vortexed gently and incubated for 5 minutes at room temperature. The formed particles-protein complexes were precipitated by addition of acetonitrile to a final concentration of 70% [V/V], mixed briefly before incubating for 18 minutes at room temperature. Particles were then immobilized using a magnetic rack (DynaMag-2 Magnet; Thermo Fisher Scientific) and supernatant was discarded. SDS was removed by washing two times with 200 μl 70% ethanol and one time with 180 μl 100% acetonitrile. After removal of organic solvent, particles were resuspended in 100 μl of 50 mM NH_4_HCO_3_ and samples digested by incubating with 1 μg of Trypsin overnight at 37°C. Samples were acidified to a final concentration of 1% Trifluoroacetic acid (Uvasol; MERCK KgaA) prior to immobilizing the beads on the magnetic rack. Forty percent of each recovered digest was then desalted and concentrated using stage tips with two stacked C18 plugs (Empore; MERCK KgaA)^11^. Stage tips were activated with three times 100 µl acetonitrile and equilibrated with three times 100 µl of 0.4% formic acid, 2% TFA in water before loading the samples. Salts were cleaned up with 100 µl of 0.1% TFA and peptides were eluted using two times 50 µl 90% acetonitrile, 0.4% formic acid. Finally, eluates were dried in a vacuum concentrator and reconstituted in 10 µl of 0.1% TFA.

Liquid chromatography tandem mass spectrometry (LC-MS/MS) was performed on a Q Exactive Hybrid Quadrupole-Orbitrap mass spectrometer (Thermo Fisher Scientific) coupled to a Dionex Ultimate 3000 RSLCnano system (Thermo Fisher Scientific) via nanoflex ion source interface. Tryptic peptides were loaded onto a trap column (Acclaim PepMap 100 C18, 3μm, 5 × 0.3 mm; Thermo Fisher Scientific) at a flow rate of 10 μl/min using 0.1% TFA as loading buffer. After loading, the trap column was switched in-line with a 50 cm, 75 µm inner diameter analytical column (packed in-house with ReproSil-Pur 120 C18-AQ, 3 μm; Dr. Maisch). Mobile-phase A consisted of 0.4% formic acid in water and mobile-phase B of 0.4% formic acid in a mix of 90% acetonitrile and 10% water. The flow rate was set to 230 nl/min and a 90 min gradient applied (4 to 24% solvent B within 82 min, 24 to 36% solvent B within 8 min and, 36 to 100% solvent B within 1 min, 100% solvent B for 6 min before re-equilibrating at 4% solvent B for 18 min).

For the MS/MS experiment, the Q Exactive MS was operated in a top 10 data-dependent acquisition mode with a MS1 scan range of 375 to 1,650 m/z at a resolution of 70,000 (at 200 m/z). Automatic gain control (AGC) was set to a target of 10^6^ and a maximum injection time of 55 ms. MS^2^-scans were acquired at a resolution of 15,000 (at 200 m/z) with AGC settings of 10^5^ and a maximum injection time of 110 ms. Precursor isolation width was set to 1.6 Da and the HCD normalized collision energy to 28%. The threshold for selecting precursor ions for MS2 was set to ~2,000 counts. Dynamic exclusion for selected ions was 90 sec. A single lock mass at m/z 445.120024 was employed^12^, XCalibur version 4.3.73.11 and Tune 2.11.3006 were used to operate the instrument.

Acquired raw data files were processed using the Proteome Discoverer 2.4.1.15 platform, utilizing the database search engine Sequest HT. Percolator V3.0 was used for validation and results filtered with a false discovery rate (FDR) of 1% on PSM, peptide and protein level under strict conditions. Searches for bee venom samples were performed with full tryptic digestion against the *Apis mellifera* UniProtKB data base v2020.06 (17,151 sequences and appended known contaminants) and *Apis mellifera* SwissProt database v2020.06 (73 sequences) with up to two miscleavage sites. Hu PBCMC supernatant samples were searched against the human SwissProt database v2020.06 (20,395 sequences and appended known contaminants) or in combination with *Apis mellifera* databases for bee venom treated with hu PBCMC supernatant with up to two miscleavage sites. Oxidation (+15.9949 Da) of methionine and acetylation of protein N-terminus (+42.0110 Da) were set as variable modifications, whilst carbamidomethylation (+57.0214 Da) of cysteine residues was set as a fixed modification. Data was searched with mass tolerances of ±10 ppm and 0.025 Da on the precursor and fragment ions, respectively. Results were filtered to include peptide spectrum matches (PSMs) with Sequest HT cross-correlation factor (Xcorr) scores of ≥1, proteins with more than 1 unique peptide, and quant values detected in at least all samples. For calculation of protein intensities Minora Feature Detector node and Precursor Ions Quantifier node, both integrated in Thermo Proteome Discoverer, were used. Automated chromatographic alignment and feature linking mapping were enabled with the total peptide amount used for normalization between individual runs.

The mass spectrometry data (of BV, hu PBCMC supernatant, and the combination of both) have been deposited to the ProteomXchange Consortium via the PRIDE partner repository^13^ with the dataset identifier PXD023851 and 10.6019/PXD023851**.**

Exported data was then analyzed using R version 4.0.3. Proteins not found in any of the *Apis mellifera* databases were excluded. Additionally, one of the detected peptide sequences matched to *Apis mellifera* superoxide dismutase also matched to *Homo sapiens* superoxide dismutase. Since this protein was detected with higher abundance in MC SN-treated BV, we assume that this represents a false positive match (secreted by activated hu PBCMCs) and excluded it from further analysis. The remaining abundances were log_2_-transformed and differential abundance of proteins was analyzed using the *limma* package^14^. Features with an absolute log_2_ fold change > 1 and FDR < 0.1 were considered differentially abundant.

### PLA_2_ activity assay

For assessment of PLA_2_ activity, 5x 10^5^ FSCMCs were sensitized with 1 µg/ml anti-DNP IgE overnight. The next day, FSCMCs were washed and resuspended in RPMI without phenol red at 4x 10^6^ cells/ml. Cells were then stimulated 1 h at 37°C by addition of an equal volume of 20 ng/ml DNP-HSA, followed by centrifugation and harvesting of the supernatant. Twenty-five µl of undiluted (equivalent to compounds secreted by 50000 cells) or serially 1:10 diluted supernatant or Heparin solution were incubated with 25 µl of 500 µg/ml BV (total of 12.5 µg) and were mixed in a 96 well v-bottom plate and incubated 10 min at 37°C. Next, 100 µl of substrate supernatant (see below) was added, mixed and incubated for 30 minutes at 37°C. The plate then was centrifuged for 2 minutes at 800 g and 100 µl of supernatant was transferred to a 96 well flat bottom plate. Absorbance at 425 nm (with the 600 nm reference value subtracted) was measured using a Sunrise microplate reader (Tecan).

The substrate for analysis of PLA_2_ activity^15,16^ was prepared fresh: 4N3OBA (stored 250 mM in DMSO at -20°C) was diluted in 1x substrate buffer (10 mM TRIS HCl, 10 mM CaCl_2_, 100 mM NaCl, pH 8.0) to a final concentration of 1 mM, vortexed thoroughly and centrifuged 5 minutes at 18000 g. The substrate supernatant was used for enzyme activity measurement (see above).

### Realtime MC degranulation assessment by flow cytometry

FSCMCs were sensitized at 5x 10^5^ cells/ml (2 ml/well/sample in a 12 well tissue culture plate) overnight 1:20 with PBS or BV serum (sera processed as described above). Next day, cells were washed with complete culture medium (without cytokines) and resuspended in 500 µl medium containing 1:500 propidium iodide (Invitrogen) and 8 µg/ml Avidin^AF488^, transferred to a FACS tube and warmed 15 minutes at 37°C. Next, pre-warmed 20 µg/ml BV in medium (or plain medium) was added to the FACS tube and immediately analyzed over 30 minutes (flowrate 25 µl/min) on an Accuri C6 flow cytometer (BD Biosciences).

### Single cell degranulation analysis by confocal fluorescence microscopy

Real-time MC degranulation analysis by confocal fluorescence microscopy was performed as previously described with minor modification^7^. Briefly, eight-well Lab-Tek 1.0 borosilicate cover glass chamber slides (Nunc) were coated with 5 µg/ml poly-D-lysine in PBS. FSCMCs were sensitized at 5x 10^5^ cells/ml (2 ml/well/sample in a 12 well tissue culture plate) overnight 1:20 with PBS or BV serum (sera processed as described above). The next day, cells were washed with complete culture medium (without cytokines) and resuspended in 500 µl medium containing 2 µM Fluo-4 AM (Invitrogen) and incubated for 30 min at 37°C. Cells were then washed twice with medium and resuspended at 2.5x 10e5/ml in medium containing 25 µg/ml avidin^SR^ and 5 µM TO-PRO-3 (viability stain; Thermo Fisher Scientific) and 190 µl suspension were seeded to a coated-chamber slide well, and transferred to the pre-warmed Zeiss LSM780 inverted confocal laser-scanning microscope. Cells were allowed to settle and adhere for 10 minutes, followed by addition of 10 µl of 200 µg/ml BV (or medium) and immediate time lapse recording of fluorescence for 1 hour. Images were processed and exported using Zen software (Zeiss) and mean fluorescence intensity (MFI) and mean integrated intensity (MII) were quantified as previously described using Image J/FIJI software^7,17^.

### Venom cytotoxicity analysis

Twenty-thousand 3T3-Swiss albino fibroblasts (ATCC CCL-92) were seeded in poly-D-lysine coated 8 well chamber slides (see above) in DMEM medium supplemented with 10% FBS, 1x penicillin/streptomycin and 1x glutamine (all from Gibco). The next day, 20 µl MC supernatant (prepared in RPMI w/o PR as described above in section BV digest analysis) was incubated either with 10 µl protamine chloride (protamine; 400 µg/ml RPMI w/o PR) or 10 µl 4x protease inhibitor (and supplemented with RPMI w/o PR to total 30 µl) for 20 minutes at room temperature. Next, 10 µl of BV (800 µg/ml) was added and incubated for 30 minutes at 37°C. In parallel, fibroblasts were stained with 2 µM Fluo-4 AM in RPMI w/o PR 10% FBS for 30 minutes at 37°C. Cells were washed twice with medium and 225 µl RPMI w/o PR 10% FBS with 5 µM TO-PRO-3 was added and cells were moved to the pre-warmed microscope (see above). After approximately 10 minutes, 25 µl of untreated or treated BV (resulting in a final concentration 20 µg/ml) was added, followed by immediate time lapse recording of fluorescence for 1 hour.

In experiments assessing the toxicity of BV in combination with protamine and protease inhibitor (Fig. S4B), BV was mixed 1:1 with RPMI w/o PR, 400 µg/ml protamine or 4x protease inhibitor, followed by transfer of 25 µl of the BV mix to chamber slides with Fluo-4 AM-stained 3T3 fibroblasts in 225 µl RPMI w/o PR 10% FBS with 5 µM TO-PRO-3 addition, resulting in final BV concentrations of 20 µg/ml, 10 µg/ml or 5 µg/ml (with constant optional protamine and protease inhibitor concentrations). Images of the same field of view were taken before and 60 minutes after addition of the BV mix.

Images were processed and exported using Zen software (Zeiss) and mean TO-PRO-3 fluorescence intensity (MFI) was quantified as previously described using Image J/FIJI software^7,17^.

### BV-mediated ear swelling

C57BL/6 mice were anesthetized and intradermally injected with either 20 µl PBS serum or BV serum into both ears. The next day, 20 µl PBS alone or PBS containing 200 ng BV were injected into one ear, respectively, of each mouse. Ear thickness was measured using a 0.01 mm Dial Thickness Gauge (Peacock Ozaki) directly before and at indicated timepoints after injection.

### *In vivo* imaging

*Mcpt5-Cre^cre^; R26Y^EYFP^* mice were intradermally injected with 20 µl of PBS or BV serum into the ear. Next day the mice were intradermally injected with 20 µl PBS containing 10 µg/ml BV and 8 µg of avidin^SR^. Ears were assessed by intravital 2 photon microscopy 30 minutes later and MC degranulation was analyzed as previously described^7^.

### Analysis and software

Flow cytometry data were analyzed using FlowJo (FlowJo LLC). Confocal microscopy data were processed and analyzed using Zen software (Zeiss) and Image J/FIJI^17^. Two-photon microscopy data were analyzed using Imaris software (Oxford Instruments)^7^. Statistical analyses (apart from that of mass spectrometry results; see above) were conducted using GraphPad Prism (GraphPad Software) and tests were applied as indicated in the figure legends. Differences with *P* values equal or below 0.05 were considered significant. All experiments were performed at least twice.

## Supplementary Figure Legends

### Figure S1: BV response of FSCMCs and serum characterization

(A and B) FSCMCs were incubated with different concentrations of BV for 1 hour. (A) Cell viability assessed as % of propidium iodide (PI)-positive (dead) cells by flow cytometry (representative of two independent experiments). (B) Degranulation assessed as ß hexosaminidase (ß-hex) release into the cell supernatant (% of total ß-hex signal; representative of two independent experiments). (C) C57BL/6 wt mice were sensitized with 2 subcutaneous injections (on the back) of 50 µl PBS alone or containing 200 µg BV. Three weeks later, blood was collected and BV-specific IgG1, IgG2b and IgG2c antibody content and total IgE (in sera of individual mice) as well as BV PLA_2_-specific IgE (in serum pools), was analyzed by ELISA. Each symbol represents either serum from an individual mouse (collected from 2-3 immunization experiments) or a serum pool (collected in 3-4 independent immunization experiments). (A and B) mean +SD; (C) mean +SEM; (A and B) One-way ANOVA with Dunnett’s test for multiple comparisons (P values are adjusted for multiple testing); * (or n.s. – not significant) indicates comparisons with the untreated (0 µg/ml BV) group; P values are adjusted for multiple testing; (C) Mann-Whitney test; n = 20-40 (individual animals) or 3-4 (serum pools); * *P* ≤ 0.05; *** *P* ≤ 0.001

### Figure S2: Phospho-flow cytometry profile of IgE and BV-stimulated FSCMCs

(A) FSCMCs were sensitized overnight with anti-DNP IgE and not stimulated or simulated with either 10 µg/ml BV, 5 ng/ml DNP-HSA, or both. Phosphorylation of AKT, MAPK/ERK and PLCγ1 and membrane-localized Lamp-1 was measured before (0 min) and after stimulation for 1, 5, 10 and 30 minutes by (phospho-) flow cytometry. Selected histogram overlays (upper panels) and respective mean fluorescence intensities (MFIs, lower panels) of phosphorylation data 5 minutes after stimulation, as shown in Figure 3C. Data are from one of three independent experiments, each of which gave similar results.

### Figure S3: PAGE analysis of BV and purified BV toxins

(A) Complete BV (40 µg; lane 2), purified BV PLA_2_ (20 µg; lane 3) or purified BV melittin (20 µg; lane 4) were separated by PAGE (15% acrylamide), followed by Coomassie blue staining of the gel. Labels on the left of the picture indicate the molecular weight of the protein marker (lane 1) band at the same height (or as indicated by a connecting line). The experiment was performed once.

### Figure S4: Viability of 3T3 fibroblasts exposed to FSCMC supernatant, protamine or protease inhibitor

(A) 3T3 fibroblasts were seeded in chamber slides and stained with Fluo-4 (green) and left untreated or exposed to supernatant of IgE/antigen (DNP-HSA)-activated FSCMCs that were either untreated or pre-treated with protamine or protease inhibitor (as shown in Figure 4D). Cell death was monitored by confocal fluorescence microscopy imaging of TO-PRO-3 (turquoise)-positive nuclei over 60 minutes. Representative pictures (outermost left: merge of brightfield, Fluo-4 and TO-PRO-3; remaining pictures show merges of Fluo-4 and TO-PRO-3 channels) of the field of views at different timepoints after treatment are shown. (B) 3T3 fibroblasts were seeded in chamber slides and stained with Fluo-4 (green) and incubated for 60 minutes with 5, 10 or 20 µg/ml BV only or BV in combination with protamine (100 µg/ml)- or protease inhibitor (1x final concentration) in the presence of TO-PRO-3. Cell death was monitored by confocal fluorescence microscopy immediately before (0 min) and 60 minutes after addition of BV (and additives) by imaging of TO-PRO-3 (turquoise)-positive nuclei. Representative pictures (merges of Fluo-4 and TO-PRO-3 channels) of the field of views at the respective timepoints are shown. The lower panels illustrate the proportions of dead (with MFI ≥ 1) and live (with MFI < 1) cells (numbers indicate the respective identified and quantified nuclei) in the field of view after 60 minutes of BV exposure (cells with a TO-PRO-3-positive nucleus at timepoint 0 min were excluded from the analysis). Turquoise stains label nuclei of dead cells. See also Figure 4D and 6A. (A and B) The scale bars represent 10 µm. Data are representative of 2 independent experiments.

### Figure S5: Response of hu PBCMCs BV-mediated activation, and BV degradation

(A and B) Human peripheral blood-derived cultured MCs (hu PBCMCs) were stimulated with the indicated concentrations of BV and analyzed after 1 hour. (A) Tryptase activity in the supernatant (% of total tryptase-mediated signal). (B) Percentage of avidin-positive cells among PI-negative (live) cells. (C) Ten µg BV were exposed for 1 hour to supernatant (SN) collected different numbers of IgE/anti-IgE stimulated (for 1 hour) hu PBCMCs, followed by PAGE and staining of the gel with Coomassie blue. Labels on the left of the picture indicate the molecular weight of the protein marker (not depicted) band at the respective height. (D) Heatmap depicting mass spectrometry analysis results (of sample triplicates) of BV protein abundance after 1 h exposure to SN of IgE/anti-IgE-stimulated (1 hour) hu PBCMCs (*vs*. untreated BV). All 118 detected BV-specific proteins are shown. Characterized allergens (Api m 1-12) are indicated. Raw abundances were log2- and z-score-transformed prior to visualization. Raw and adjusted P values and fold changes are shown in Table S1. (A-C) Data are from one of at least two independent experiments, each of which gave similar results. (A and B) graphs depict mean + SD. One-way ANOVA with Dunnett’s test for multiple comparisons (P values are adjusted for multiple testing); * (or n.s. – not significant) indicates comparisons with the untreated (0 µg/ml BV) group; P values are adjusted for multiple testing; ****P* ≤ 0.001 (D) Mass spectrometry analysis of sample triplicates was performed once.

## Supplementary Tables

### Table S1: Mass spectrometry analysis of BV exposed to supernatant of IgE/anti-IgE-activated hu PBCMCs

Human peripheral blood-derived cultured MCs (hu PBCMCs) were stimulated with IgE/anti-IgE (for 1 hour) and the supernatant (MC SN) was collected. BV was exposed in triplicate (rep_1 – 3) for 1 h to MC SN (BV+MC SN) or PBS (untreated) and analyzed by mass spectrometry. Normalized abundance and P values were calculated as described in the methods section. Data in the table correspond to results shown in Fig. 5E and F and S5D.

## Supplementary Videos

### Videos S1-3: Time lapse of the IgE-mediated FSCMC degranulation response to BV

FSCMCs were incubated overnight with isotype control-pre-treated PBS serum or isotype control- or anti-IgE-pre-treated BV serum. The next day, cells were seeded in chamber slides stained with Fluo-4 and exposed to 10 µg/ml BV in presence of avidin^SR^ and To-Pro-3. The avidin^SR^-mediated signal of degranulating MCs was recorded over 60 minutes by realtime confocal fluorescence microscopy. The video shows a time-lapse of merged fluorescence signals of Fluo-4 (calcium, in green) To-Pro-3 (viability, in turquoise) and avidin^SR^ (in red). Turquoise stains are nuclei of dead cells. Video S1: PBS serum, isotype ctrl, 10 µg/ml BV; Video S2: BV serum, isotype ctrl, 10 µg/ml BV; and Video S3: BV serum, anti-IgE, 10 µg/ml BV.

### Videos S4-7: Realtime monitoring of FSCMC-mediated BV detoxification

3T3 fibroblasts were seeded in chamber slides and stained with Fluo-4 (green). BV was optionally pre-treated with supernatant of IgE/BV-antigen-activated (BV serum/10 µg/ml BV; stimulated for 1 hour) FSCMCs that were either untreated or pre-treated with protamine or protease inhibitor. The untreated or pre-treated BV was transferred onto the fibroblasts and monitoring of cell death, by confocal fluorescence microscopy imaging of To-Pro-3 (turquoise)-positive nuclei over 60 minutes, was started immediately. The video shows a time-lapse of merged fluorescence signals of Fluo-4 (calcium, in green) and To-Pro-3 (viability, in turquoise). Turquoise stains are nuclei of dead cells. Video S4: 20 µg/ml BV pre-incubated with supernatant of non-stimulated FSCMCs; Video S5: 20 µg/ml BV pre-incubated with untreated supernatant of BV-stimulated FSCMCs; Video S6: 20 µg/ml BV pre-incubated with protamine-treated supernatant of BV-stimulated FSCMCs; and Video S7: 20 µg/ml BV pre-incubated with protease inhibitor-treated supernatant of BV-stimulated FSCMCs.

### Videos S8-11: Realtime monitoring of hu PBCMC-mediated BV detoxification

3T3 fibroblasts were seeded in chamber slides and stained with Fluo-4 (green). BV was optionally pre-treated with supernatant of IgE/anti-IgE activated (stimulated for 1 hour) hu PBCMCs that were either untreated or pre-treated with protamine or protease inhibitor. The untreated or pre-treated BV was transferred onto the fibroblasts and monitoring of cell death, by confocal fluorescence microscopy imaging of To-Pro-3 (turquoise)-positive nuclei over 60 minutes, was started immediately. The video shows a time-lapse of merged fluorescence signals of Fluo-4 (calcium, in green) and To-Pro-3 (viability, in turquoise). Turquoise stains are nuclei of dead cells. Video S8: 20 µg/ml BV pre-incubated with supernatant of non-stimulated hu PBCMCs; Video S9: 20 µg/ml BV pre-incubated with untreated supernatant of IgE/anti-IgE-stimulated hu PBCMCs; Video S10: 20 µg/ml BV pre-incubated with protamine-treated supernatant of IgE/anti-IgE-stimulated hu PBCMCs; and Video S11: 20 µg/ml BV pre-incubated with protease inhibitor-treated supernatant of IgE/anti-IgE-stimulated hu PBCMCs.

# Supplementary References

1. Scholten J, Hartmann K, Gerbaulet A, et al. Mast cell-specific Cre/loxP-mediated recombination in vivo. *Transgenic Res.* 2008;17(2):307-315.

2. Srinivas S, Watanabe T, Lin CS, et al. Cre reporter strains produced by targeted insertion of EYFP and ECFP into the ROSA26 locus. *BMC Dev Biol.* 2001;1:4.

3. Liu FT, Bohn JW, Ferry EL, et al. Monoclonal dinitrophenyl-specific murine IgE antibody: preparation, isolation, and characterization. *J Immunol.* 1980;124(6):2728-2737.

4. Marichal T, Starkl P, Reber LL, et al. A beneficial role for immunoglobulin E in host defense against honeybee venom. *Immunity.* 2013;39(5):963-975.

5. Starkl P, Marichal T, Gaudenzio N, et al. IgE antibodies, FcepsilonRIalpha, and IgE-mediated local anaphylaxis can limit snake venom toxicity. *J Allergy Clin Immunol.* 2016;137(1):246-257 e211.

6. Meindl S, Schmidt U, Vaculik C, Elbe-Burger A. Characterization, isolation, and differentiation of murine skin cells expressing hematopoietic stem cell markers. *J Leukoc Biol.* 2006;80(4):816-826.

7. Gaudenzio N, Sibilano R, Marichal T, et al. Different activation signals induce distinct mast cell degranulation strategies. *The Journal of clinical investigation.* 2016;126(10):3981-3998.

8. Akahoshi M, Song CH, Piliponsky AM, et al. Mast cell chymase reduces the toxicity of Gila monster venom, scorpion venom, and vasoactive intestinal polypeptide in mice. *The Journal of clinical investigation.* 2011;121(10):4180-4191.

9. Pejler G, Hu Frisk JM, Sjostrom D, Paivandy A, Ohrvik H. Acidic pH is essential for maintaining mast cell secretory granule homeostasis. *Cell Death Dis.* 2017;8(5):e2785.

10. Hughes CS, Foehr S, Garfield DA, Furlong EE, Steinmetz LM, Krijgsveld J. Ultrasensitive proteome analysis using paramagnetic bead technology. *Mol Syst Biol.* 2014;10:757.

11. Rappsilber J, Mann M, Ishihama Y. Protocol for micro-purification, enrichment, pre-fractionation and storage of peptides for proteomics using StageTips. *Nat Protoc.* 2007;2(8):1896-1906.

12. Olsen JV, de Godoy LM, Li G, et al. Parts per million mass accuracy on an Orbitrap mass spectrometer via lock mass injection into a C-trap. *Mol Cell Proteomics.* 2005;4(12):2010-2021.

13. Perez-Riverol Y, Csordas A, Bai J, et al. The PRIDE database and related tools and resources in 2019: improving support for quantification data. *Nucleic Acids Res.* 2019;47(D1):D442-D450.

14. Ritchie ME, Phipson B, Wu D, et al. limma powers differential expression analyses for RNA-sequencing and microarray studies. *Nucleic Acids Res.* 2015;43(7):e47.

15. Oliveira SC, Fonseca FV, Antunes E, et al. Modulation of the pharmacological effects of enzymatically-active PLA2 by BTL-2, an isolectin isolated from the Bryothamnion triquetrum red alga. *BMC Biochem.* 2008;9:16.

16. Holzer M, Mackessy SP. An aqueous endpoint assay of snake venom phospholipase A2. *Toxicon.* 1996;34(10):1149-1155.

17. Schindelin J, Arganda-Carreras I, Frise E, et al. Fiji: an open-source platform for biological-image analysis. *Nat Methods.* 2012;9(7):676-682.
