# Supplementary figures and images for: IgE antibodies increase honeybee venom responsiveness and detoxification efficiency of mast cells
Source: Allergy. Author manuscript; Available in PMC 2023 Feb 1. (PMC8502784; doi:10.1111/all.14852)

Figure S1

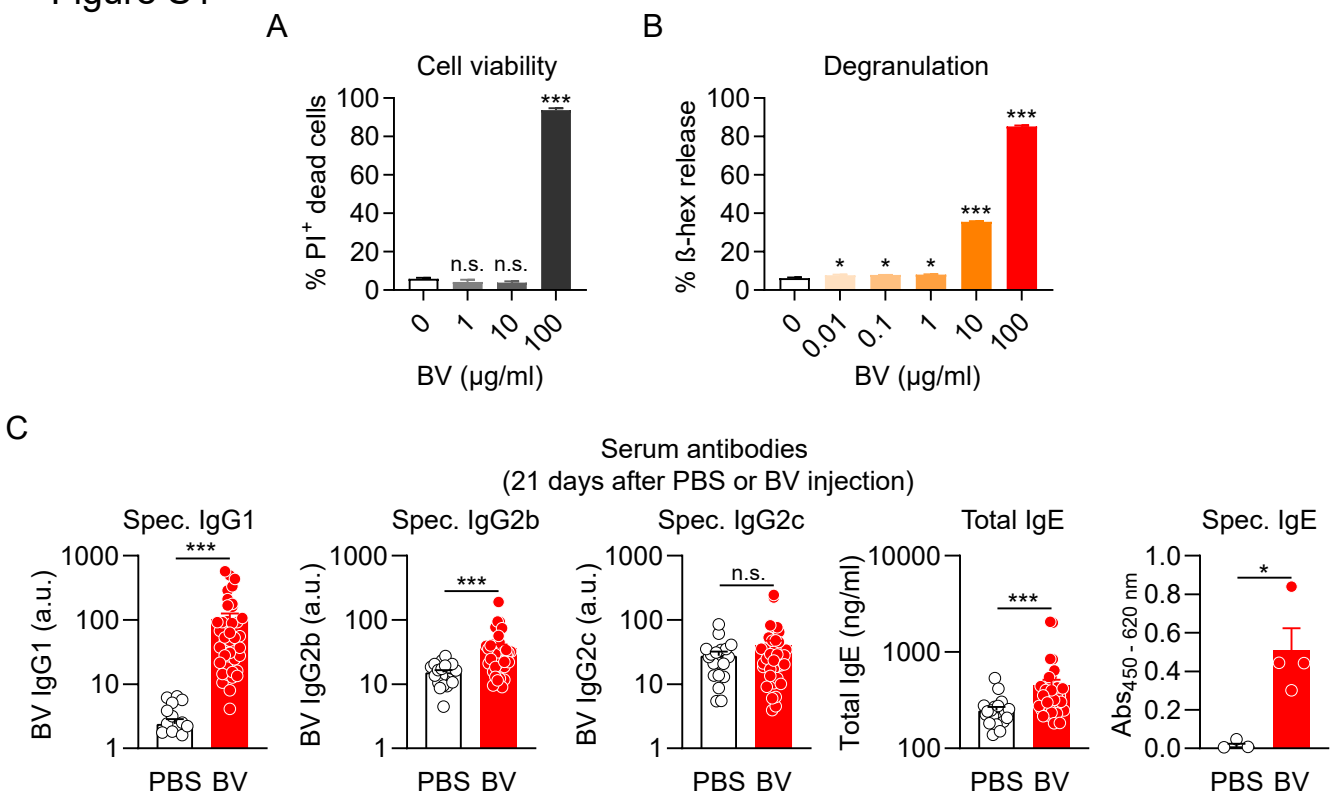

Supplement: sm14 [file NIHMS1707162-supplement-sm14.pdf]

Figure S2

A

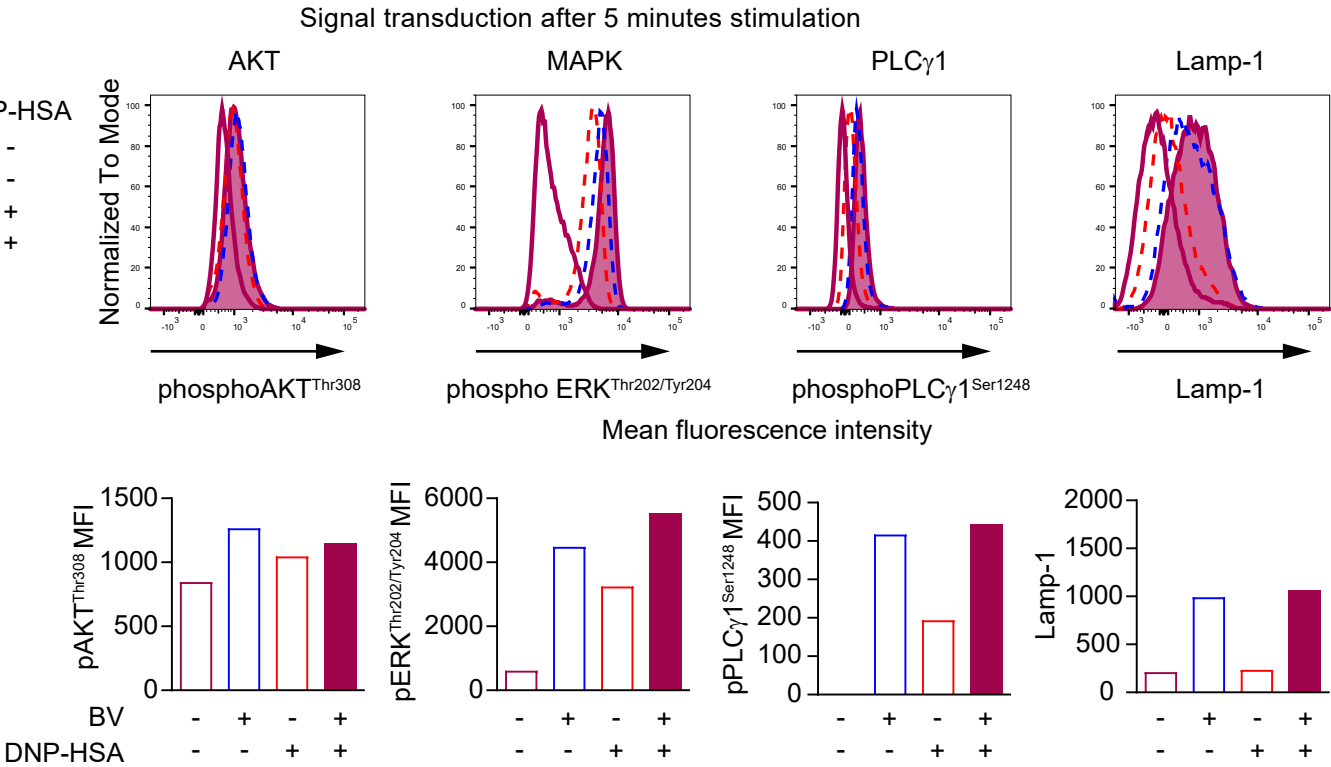

Supplement: sm15 [file NIHMS1707162-supplement-sm15.pdf]

Figure S5

A

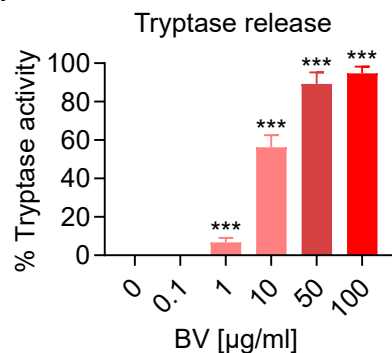

B

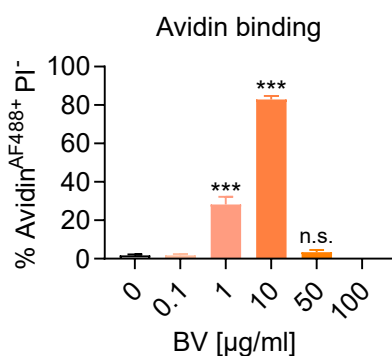

C

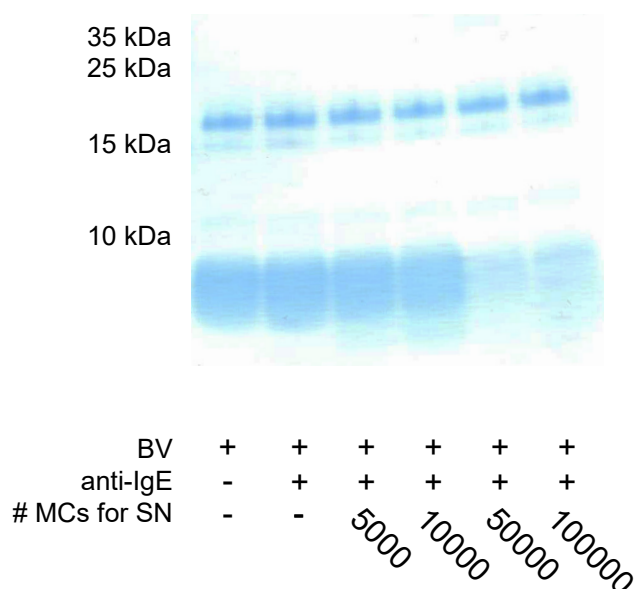

D

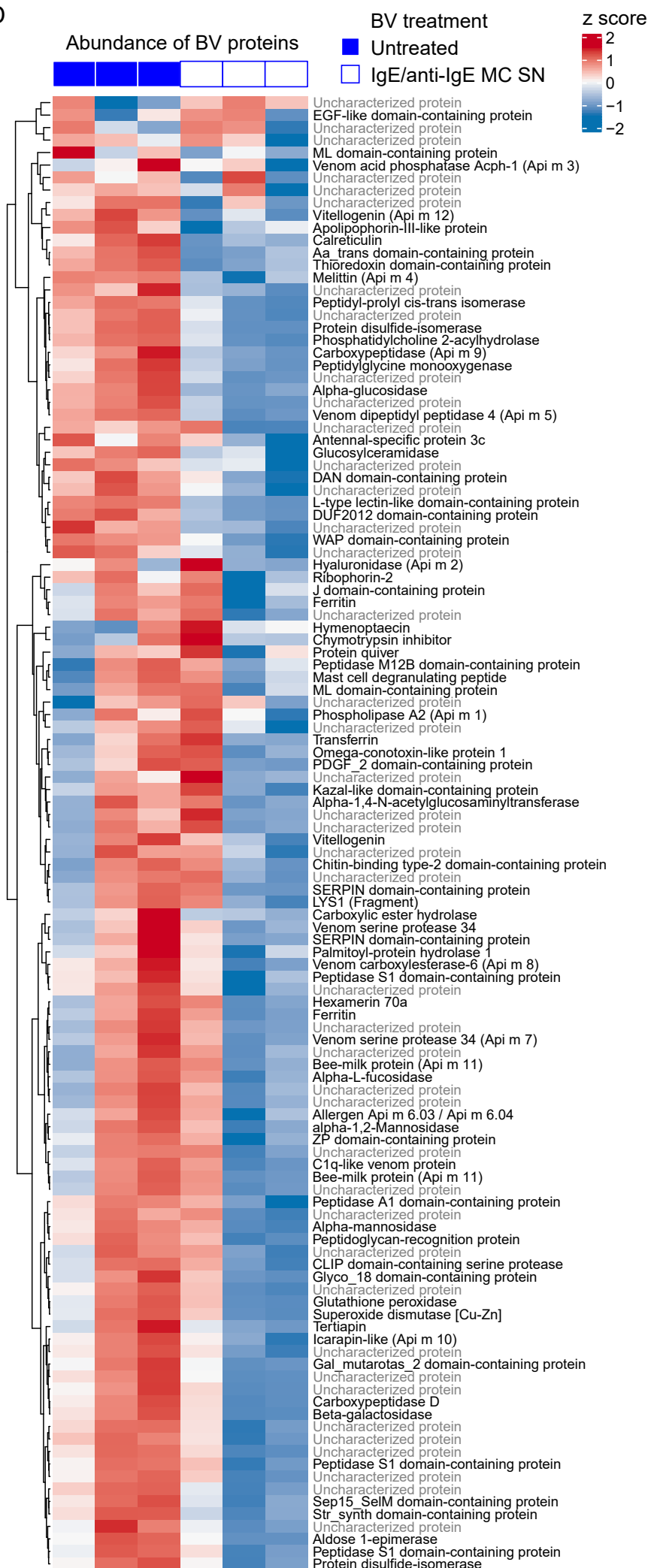

Supplement: sm18 [file NIHMS1707162-supplement-sm18.pdf]
