# Supplementary material for: IgE antibodies increase honeybee venom responsiveness and detoxification efficiency of mast cells
Source: Allergy. Author manuscript; Available in PMC 2023 Feb 1. (PMC8502784; doi:10.1111/all.14852)
Supplement: sm16 [file NIHMS1707162-supplement-sm16.pdf]

Figure S3

A

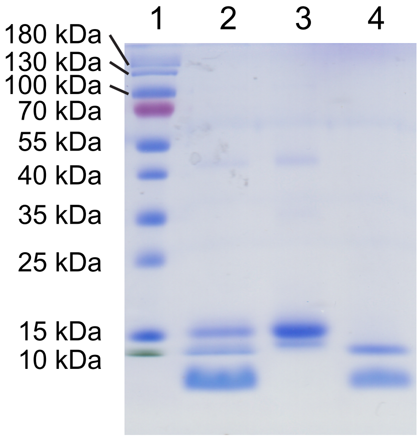


Loading scheme:
Lane 1: Protein marker
Lane 2: Complete bee venom (40 µg)
Lane 3: Purified $PLA_2$ (20 µg)
Lane 4: Purified melittin (20 µg)
