# Supplementary material for: IgE antibodies increase honeybee venom responsiveness and detoxification efficiency of mast cells
Source: Allergy. Author manuscript; Available in PMC 2023 Feb 1. (PMC8502784; doi:10.1111/all.14852)
Supplement: sm17 [file NIHMS1707162-supplement-sm17.pdf]

Figure S4

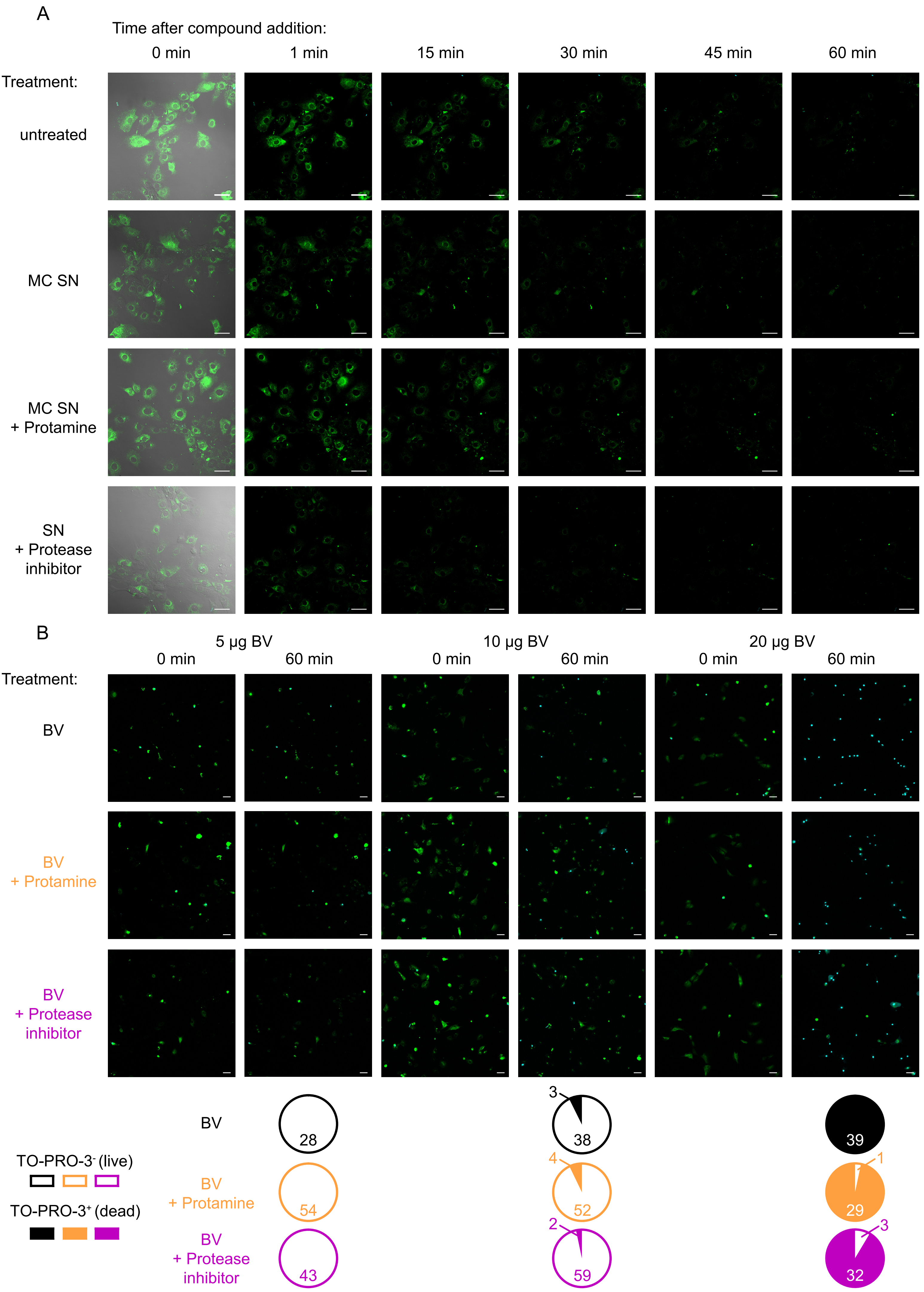
A
Time after compound addition:
0 min
1 min
15 min
30 min
45 min
60 min
Treatment:
untreated
MC SN
MC SN
+ Protamine
SN
+ Protease
inhibitor
B
5 µg BV
10 µg BV
20 µg BV
0 min
60 min
Treatment:
BV
BV
+ Protamine
BV
+ Protease
inhibitor
BV
28
3
38
39
TO-PRO-3⁻ (live)
BV
+ Protamine
54
4
52
1
29
TO-PRO-3⁺ (dead)
BV
+ Protease
inhibitor
43
2
59
3
32
